# Occurrence of excited state charge separation in a N-doped graphene–perylenediimide hybrid formed *via* ‘click’ chemistry†

**DOI:** 10.1039/c9na00416e

**Published:** 2019-08-30

**Authors:** Habtom B. Gobeze, Luis M. Arellano, Ana María Gutiérrez-Vílchez, María J. Gómez-Escalonilla, Ángela Sastre-Santos, Fernando Fernández-Lázaro, Fernando Langa, Francis D'Souza

**Affiliations:** Department of Chemistry, University of North Texas 1155 Union Circle, #305070 76203-5017 Denton TX USA Francis.DSouza@UNT.edu; Universidad de Castilla-La Mancha, Instituto de Nanociencia, Nanotecnología y Materiales Moleculares (INAMOL) 45071-Toledo Spain Fernando.Langa@uclm.es; Área de Química Orgánica, Instituto de Bioingeniería, Universidad Miguel Hernández Avda. de la Universidad, s/n Elche 03202 Spain fdofdez@umh.es

## Abstract

Hetero-atom doped graphene is a two-dimensional material with a band gap, needed to build optoelectronic devices. However, research progress in this area has been sluggish due to synthetic challenges to build energy harvesting materials, especially donor–acceptor type hybrids. In the present study, using *click* chemistry, we have successfully synthesized a donor–acceptor hybrid comprised of N-doped graphene and perylenediimide (PDI), a well-known electron-accepting photosensitizer. The TGA and XPS results revealed the attachment of the PDI moiety in the hybrid. Ground and excited state interactions were monitored by a variety of spectral and electrochemical techniques. Finally, the ability of the present donor–acceptor hybrid to undergo photoinduced charge separation from singlet excited PDI was systematically probed using femtosecond transient spectral techniques. Evidence of charge separation was possible to achieve from comparison of transient and spectroelectrochemical results. These results suggest the potential use of covalently functionalized, substitutional N-doped graphene as a functional material for building optoelectronic devices.

## Abstract 2

A donor–acceptor hybrid was devised and synthesized using N-doped graphene and perylene diimide *via click chemistry*, and the occurrence of excited state charge separation is demonstrated.

## Graphical abstract

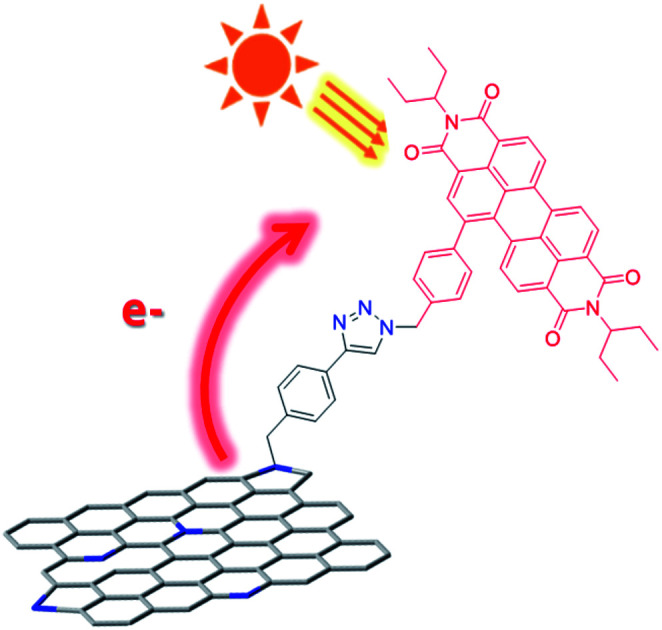

## Introduction

Graphene, a novel nanomaterial made of a single sheet of carbon atoms packed in a hexagonal lattice, has emerged as one of the most actively researched topics.^1^ The structural and electronic properties have made graphene a promising two-dimensional material for various applications, including energy storage, electrocatalysis, sensors and electronics.^2–6^ However, the lack of a band gap has significantly delayed its progress in energy harvesting and optoelectronic applications.

To tailor the properties of graphene and to introduce the much desired band gap, chemical doping has arisen as an important approach,^7–9^ which had already proven effective in the doping of carbon nanotubes greatly broadening their applications.^10,11^ There are two main methods of chemical doping of graphene, *viz.*, the adsorption (gas, metal or organic molecules) method and the substitutional doping method which introduces heteroatoms, such as nitrogen and boron atoms into the carbon lattice of graphene.^7,8^ Both methods can modulate the electronic properties of graphene while introducing a band gap.

In substitutional N-doped graphene (NG), the nitrogen atoms are incorporated into the basal plane of graphene in the forms of ‘pyridinic’, ‘pyrrolic’ and ‘graphitic’ nitrogen bonding configurations as revealed by XPS studies. The ‘pyridinic’ and ‘pyrrolic’ nitrogens mostly occur at the edge or defect sites while the ‘graphitic’ nitrogen occurs by replacing carbon in the graphene plane.^7,8^ Upon nitrogen doping in the graphene monolayer, the Fermi level shifts above the Dirac point, and the density of states near the Fermi level is suppressed, and thus, the band gap between the conduction band and the valence band is opened. Zhang *et al.*^12^ experimentally demonstrated the existence of a band gap of 0.16 eV. This remarkable property has turned the latter into an invaluable material for semiconductor devices and energy harvesting schemes.

**Fig. 1:**
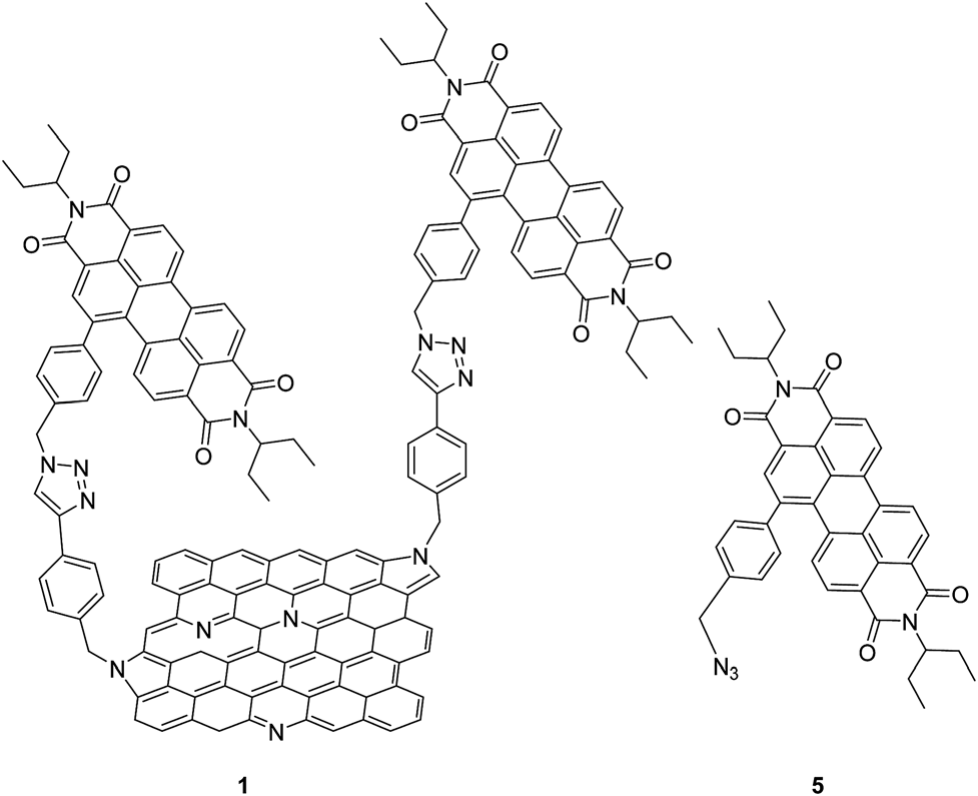
Structures of the N-doped graphene–perylenediimide hybrid, 1, and control PDI 5, investigated in the present study.

The presence of a band gap in NG could promote excited state charge transfer processes *via* oxidative or reductive electron transfer mechanisms when covalently linked to a photosensitizer-electron acceptor or donor, which is a key step for this material to be useful in light energy harvesting and photocatalysis. However, due to associated synthetic and characterization challenges, studies on such donor–acceptor hybrid materials are still in early stages.^13^ In the present study, we have made significant progress in this direction by successful covalent functionalization of a well-known electron-accepting photosensitizer, perylenediimide (PDI) to NG *via* ‘click’ chemistry (Fig. 1). Upon full characterization of this donor–acceptor hybrid, we have successfully demonstrated the occurrence of charge separation from the singlet excited state of PDI leading to radical ion-pair species using the femtosecond transient absorption (fs-TA) technique in *N*,*N*-dimethylformamide (DMF).

## Experimental

### Chemicals and materials

All chemicals were of reagent grade, purchased from commercial sources, and used as received, unless otherwise specified. All the reagents were from Aldrich Chemicals while the bulk solvents utilized in the syntheses were from Fischer Chemicals. Nitrogen-doped graphene (NG), grade TNNRGO, with a nitrogen content of 5–10% wt was supplied by Chengdu Organic Chemicals Co. Ltd., Chinese Academy of Sciences (Chengdu, China) (www.timesnano.com). Compounds 1-bromo-*N*,*N*′-bis(ethylpropyl)perylene-3,4:9,10-tetracarboxylic diimide 2^14,15^ and {4-(bromomethyl)phenyl)ethynyl)}trimethylsilane 6^16^ were synthesized according to the literature procedure. All manipulations were carried out under a dry argon atmosphere by using standard Schlenk-type techniques. Column chromatography: SiO_2_ (40–63 mm). TLC plates coated with SiO_2_ 60F_254_ were visualized using UV light.

### Instrumentation

Sample sonication was carried out by using an Elmasonic P 300 H sonicator bath (37 kHz). NMR spectra were recorded with a Bruker AC 300 and a Bruker TopSpin AV-400 spectrometer. Chemical shifts are reported in delta (*δ*) units, expressed in parts per million (ppm) downfield from tetramethylsilane (TMS) using residual protonated solvent as an internal standard {CDCl_3_, 7.26 ppm}. The ^1^H NMR splitting patterns have been described as “s, singlet; d, doublet; t, triplet and m, multiplet”. UV/vis spectra were recorded with a Helios Gamma spectrophotometer and a Shimadzu Model 2550 double monochromator UV-visible spectrophotometer. Fluorescence spectra were monitored by using a PerkinElmer LS 55 Luminescence Spectrometer and a Horiba Yvon Nanolog coupled with time-correlated single photon counting with nanoLED excitation sources. A right angle detection method was used. Mass spectra were obtained from a Bruker Microflex matrix-assisted laser desorption/ionization time of flight (MALDI-TOF) using dithranol as the matrix. FTIR spectra were recorded using a Fourier transform IR spectrophotometer (Avatar 370) using a spectral range of 4000–400 cm^−1^, with a resolution of 1 cm^−1^, and in pellets of dispersed samples of the corresponding materials in dried KBr.

Differential pulse voltammetry (DPV) was performed in ODCB/acetonitrile 4 : 1 solution. Tetrabutylammonium hexafluorophosphate (TBAPF_6_) (0.1 M) as the supporting electrolyte was purchased from Aldrich-Sigma and used without purification. Solutions were deoxygenated by argon bubbling prior to each experiment, which was run under an argon atmosphere. Experiments were done in a one-compartment cell equipped with a platinum working microelectrode (*∅* = 2 mm) and a platinum wire counter electrode. A scan rate of 0.1 V s^−1^ was used. An Ag/AgNO_3_ electrode was used as the reference and checked against the ferrocene/ferrocenium couple (Fc/Fc^+^) before and after each experiment. Spectroelectrochemical studies were performed by using a cell assembly (SEC-C) supplied by ALS Co., Ltd. (Tokyo, Japan). This assembly was comprised of a Pt counter electrode, a 6 mm Pt Gauze working electrode, and an Ag/AgCl reference electrode in a 1.0 mm path length quartz cell. The optical transmission was limited to 6 mm covering the Pt Gauze working electrode.

Thermogravimetric analyses (TGA) were performed using a TGA/DSC Linea Excellent instrument from Mettler-Toledo, under a flow of nitrogen (90 mL min^−1^). The sample (∼0.5 mg) was introduced inside a platinum crucible and equilibrated at 40 °C followed by a 10 °C min^−1^ ramp between 40 °C and 1000 °C. The weight changes were recorded as a function of temperature. X-ray Photoelectron Spectroscopy (XPS) was performed on an RBD upgraded PHI-5000C ESCA system (PerkinElmer) with Al Kα radiation (*hν* = 1486.6 eV) and the peaks were calibrated with C 1s at 284.6 eV. Raman spectra were recorded on a Renishaw inVia Raman instrument coupled with a Leica microscope at room temperature with a 514 nm exciting laser. The samples were deposited on SiO_2_ wafers. Measurements were taken with 10 s of exposure times at varying numbers of accumulations. Raman spectra images are an average of 1000 points. The laser spot was focused on the sample surface using a long working distance 100× objective. Raman spectra were collected on numerous spots on the sample and recorded with a Peltier cooled CCD camera. The intensity ratio *I*_D_/*I*_G_ was obtained by taking the peak intensities following any baseline corrections. The data were collected and analyzed with Renishaw Wire and Origin software. AFM images were recorded in tapping mode using a Multimode 8 system (Veeco Instruments Inc., Santa Barbara, USA) with a NanoScope V controller (Digital Instruments, Santa Barbara, USA) operating at room temperature under ambient air conditions. RTESP-300 Bruker silicon cantilevers with a resonance frequency of 300 kHz and a nominal force constant of 40 N m^−1^ were used for AFM measurements. The images were processed using WSxM (freely downloadable scanning probe microscopy software from http://www.wsxmsolutions.com).

Femtosecond transient absorption spectroscopy experiments were performed using an Ultrafast Femtosecond Laser Source (Libra) by Coherent incorporating diode-pumped, mode locked Ti:sapphire laser (Vitesse) and diode-pumped intracavity doubled Nd:YLF laser (Evolution) to generate a compressed laser output of 1.5 W. For optical detection, a Helios transient absorption spectrometer provided by Ultrafast Systems LLC coupled with an optical parametric amplifier (OPA) provided by Light Conversion was used. The source for the pump and probe pulses were derived from the fundamental output of Libra (compressed output 1.5 W, pulse width 100 fs) at a repetition rate of 1 kHz. 95% of the fundamental output of the laser was introduced into the OPA, while the rest of the output was used for generation of the white light continuum. In the present study, the maximum absorption wavelength for each compound was used in all the experiments. Kinetic traces at appropriate wavelengths were assembled from the time-resolved spectral data. Data analysis was performed using Surface Xplorer software supplied by Ultrafast Systems for initial analysis and later by advanced software Glotaran for target analysis. All measurements were conducted in degassed solutions at 298 K.

### Synthesis of NG–TMS

A suspension of exfoliated NG material (30 mg) in *N*-methyl-2-pyrrolidinone (NMP) (80 mL) was reacted with 4-(bromomethyl)phenyl)ethynyl)trimethylsilane 6 (3.32 g, 12.5 mmol) and K_2_CO_3_ (0.69 g, 5 mmol) for 48 hours at 70 °C under an argon atmosphere. The reaction mixture was filtered on a PTFE membrane (Millipore 0.1 μm pore) and washed several times with NMP, water, methanol, and dichloromethane (sonicated and filtered each time), until the filtered solution became colorless affording a black powder as the desired hybrid NG–TMS (40 mg).

### Synthesis of NG–PDI 1

TBAF (5 mL, 5 mmol) was added to a suspension of alkyne functionalized NG–TMS (30 mg) in NMP (42 mL) at 0 °C. The reaction was stirred at room temperature for 3 hours, filtered on a PTFE membrane (Millipore 0.1 μm pore), and then washed with NMP, MeOH and dichloromethane to obtain the deprotected NG–TMS material as a black solid, which will be used directly in the next step. PDI 5 (27 mg, 0.04 mmol), CuSO_4_·5H_2_O (3.5 mg, 0.01 mmol) and sodium ascorbate (2.8 mg, 0.01 mmol) were added to a suspension of deprotected NG–TMS (10 mg) sonicated in NMP (15 mL). The reaction mixture was degassed and stirred at 70 °C for 4 days, and then filtered on a PTFE membrane (Millipore 0.1 μm pore). In order to remove sodium ascorbate, the copper catalyst and free PDI, the black solid was washed with NMP, methanol, water and dichloromethane. The filtrate was checked by TLC and the solid was dried overnight under vacuum affording the NG–PDI 1 material as a black solid (13 mg).

## Results and discussion

**Scheme 1:**
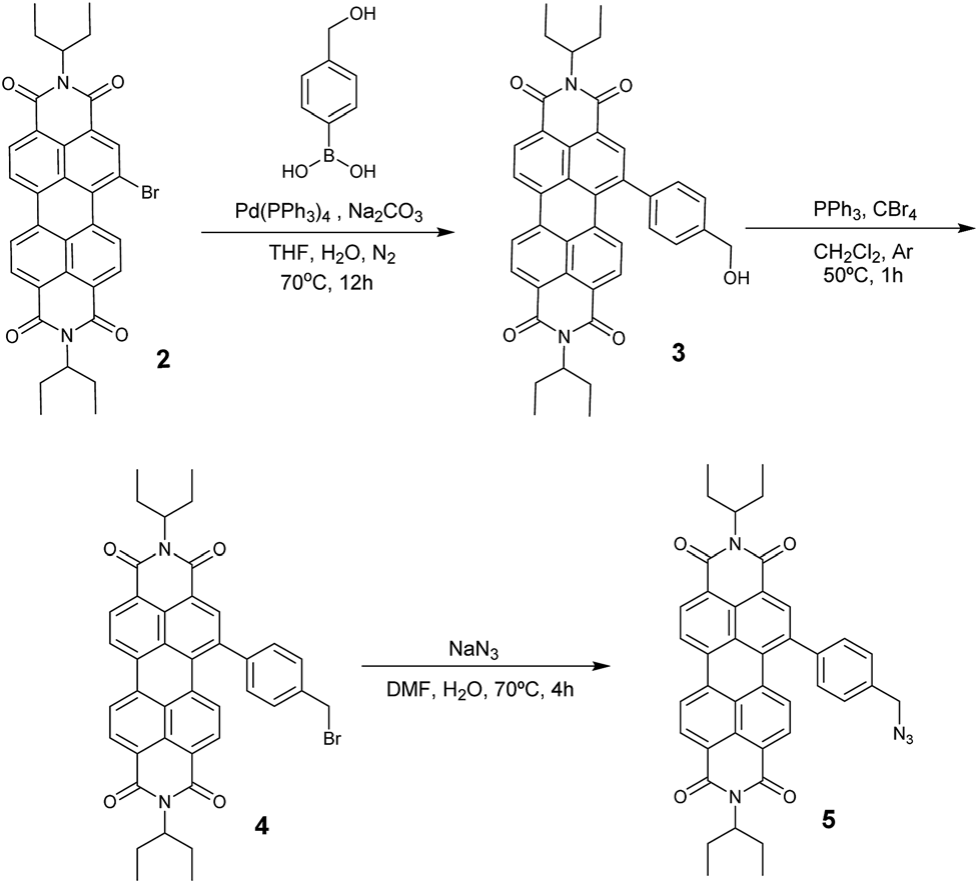
Synthetic route to PDI 5.

**Scheme 2:**
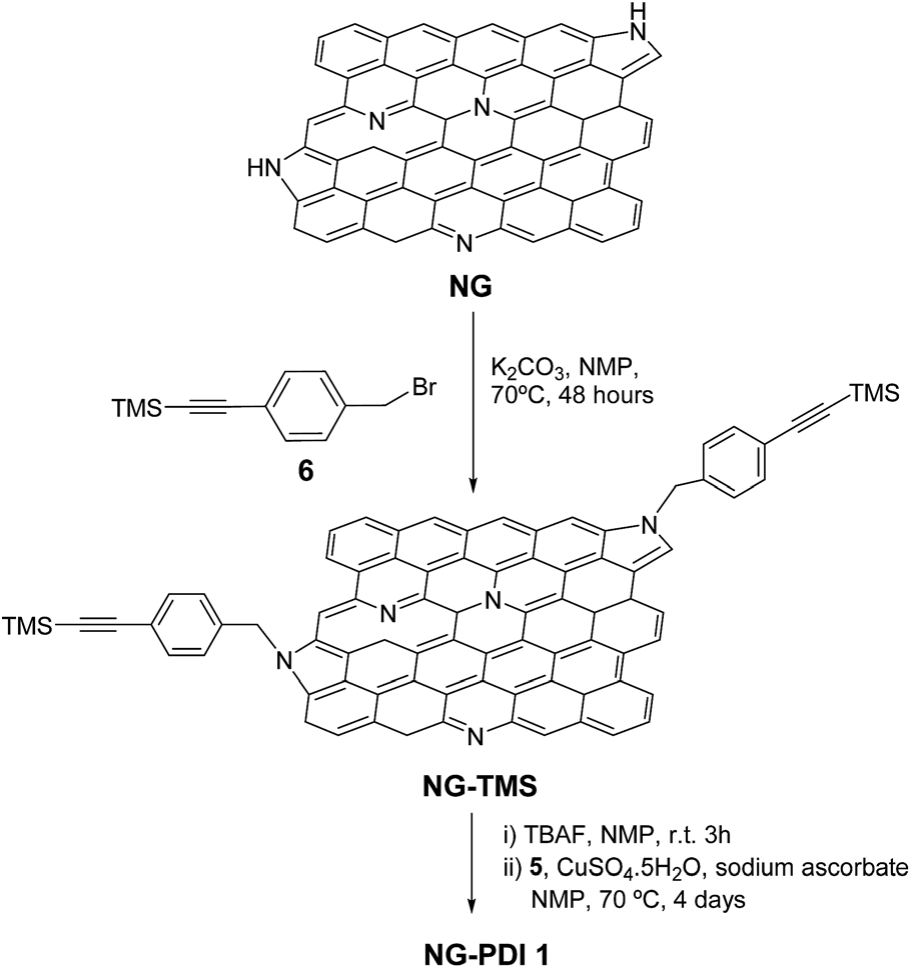
Synthesis of NG–PDI donor–acceptor hybrid 1.

Azido-substituted PDI 5 was obtained through the synthetic route depicted in Scheme 1. For this, first, PDI 3 was prepared in 87% yield by the Suzuki reaction of PDI 2 with 4-(hydroxymethyl)phenylboronic acid. Then, reaction with CBr_4_/PPh_3_ afforded PDI 4 in 46% yield, which was reacted with NaN_3_ yielding PDI 5 in 76% yield. All new compounds were characterized by ^1^H- and ^13^C-NMR, HR-MALDI-TOF and FT-IR (see ESI† for further details). The synthesis of NG–PDI hybrid 1 is shown in Scheme 2. Exfoliated NG was initially treated with {[4-(bromomethyl)phenyl]-ethynyl}trimethylsilane 6^16^ in the presence of K_2_CO_3_ obtaining functionalized NG–TMS. Subsequent treatment with tetra-*n*-butylammonium fluoride (TBAF) gave the NG derivative bearing terminal alkyne subunits, which was connected with 5 by *click* chemistry (CuSO_4_·5H_2_O and sodium ascorbate) in NMP affording the targeted NG–PDI hybrid material 1. In order to increase the solubility of hybrid 1, PDI 5 was functionalized at the N positions with swallow-tail alkyl chains.

The first evidence for the covalent functionalization of NG was obtained from thermogravimetric analysis (TGA). The TGA plot of NG–PDI hybrid 1 is shown in Fig. S1,† along with those for PDI 5, NG–TMS and the starting NG for the sake of comparison. The thermogram of NG showed a weight loss of around 10.3%, attributed to defects in the starting material; TG analysis of NG–TMS showed a loss of 32.5%; finally, hybrid 1 displayed a weight loss of 41.2% (in a temperature range of 190–550 °C); the increase with respect to NG–TMS (9%) is directly related to the decomposition of the PDI moiety, being a direct indication of covalent grafting, revealing the success of the “click” reaction adopted in the present study.

**Fig. 2:**
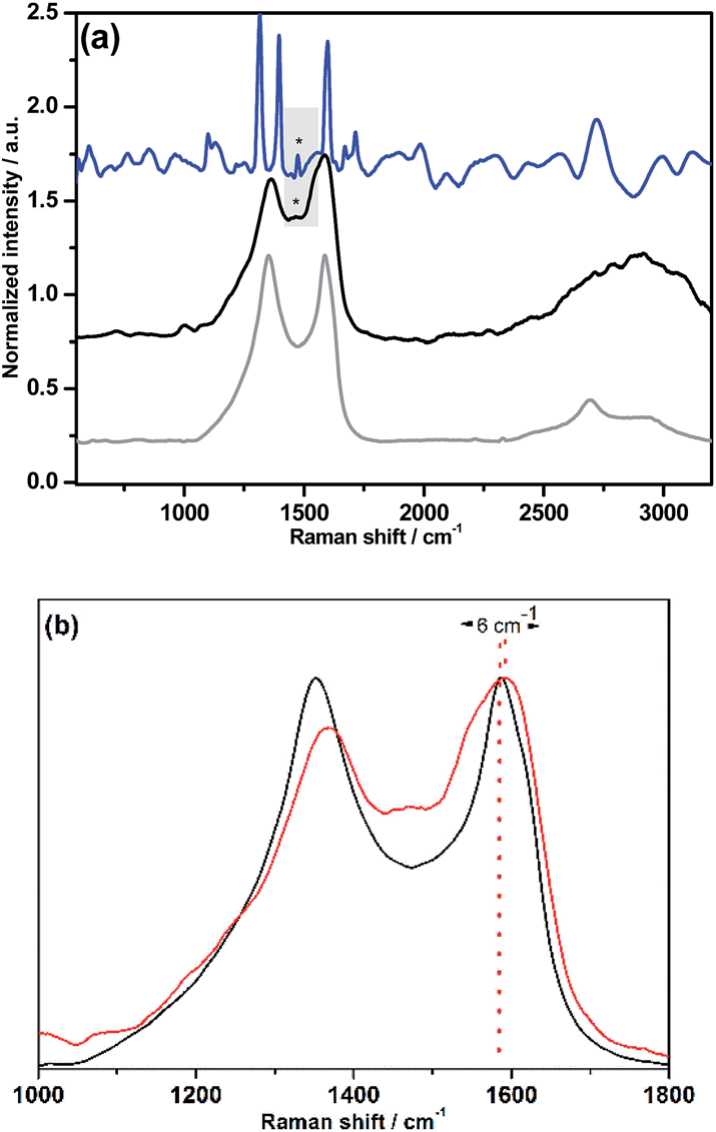
(a) Raman spectra recorded for NG (grey) compared with NG–PDI 1 (black) and the PDI precursor 5 (blue) excited at 514 nm. (b) Details of the Raman spectra from 1000 to 1800 cm^−1^ for NG (black) compared with NG–PDI 1 (red). The spectra are normalized to the intensity of the G-mode to facilitate the observation of the peak shift (3 cm^−1^).

The Raman spectrum of NG–PDI 1 contained the two most intense peaks typical for carbon nanomaterials: the D (1348 cm^−1^) and G (1590 cm^−1^) bands; evidence of the presence of the PDI group in hybrid 1 was provided by the observation of new features, especially by the broad band at 1464 cm^−1^, attributed to the perylenediimide moiety, shifted by 9 cm^−1^ compared to the band of 5, and also the broadening of the G band due to the superposition of some PDI peaks (Fig. 2a). Additionally, the G-band is upshifted by 6 cm^−1^, a finding that is consistent with the expected influence of functionalization of graphene with an electron-acceptor group (Fig. 2b).^13,17^ Importantly, no shift in the G-band was found in the case of the TMS derivative (see Fig. S2†).

To complement the characterization, FTIR spectra of NG–PDI 1 and its precursors were recorded (Fig. S3†). The band assigned to the protected alkyne group in NG–TMS disappears in hybrid 1 after the cycloaddition reaction, due to the formation of the triazole ring. Furthermore, the presence of the aliphatic C–H stretching vibration bands and the disappearance of the azide peak at 2093 cm^−1^ of PDI 5 after the “*click*” coupling confirmed that the PDI moiety was covalently bonded to the NG material.

**Fig. 3:**
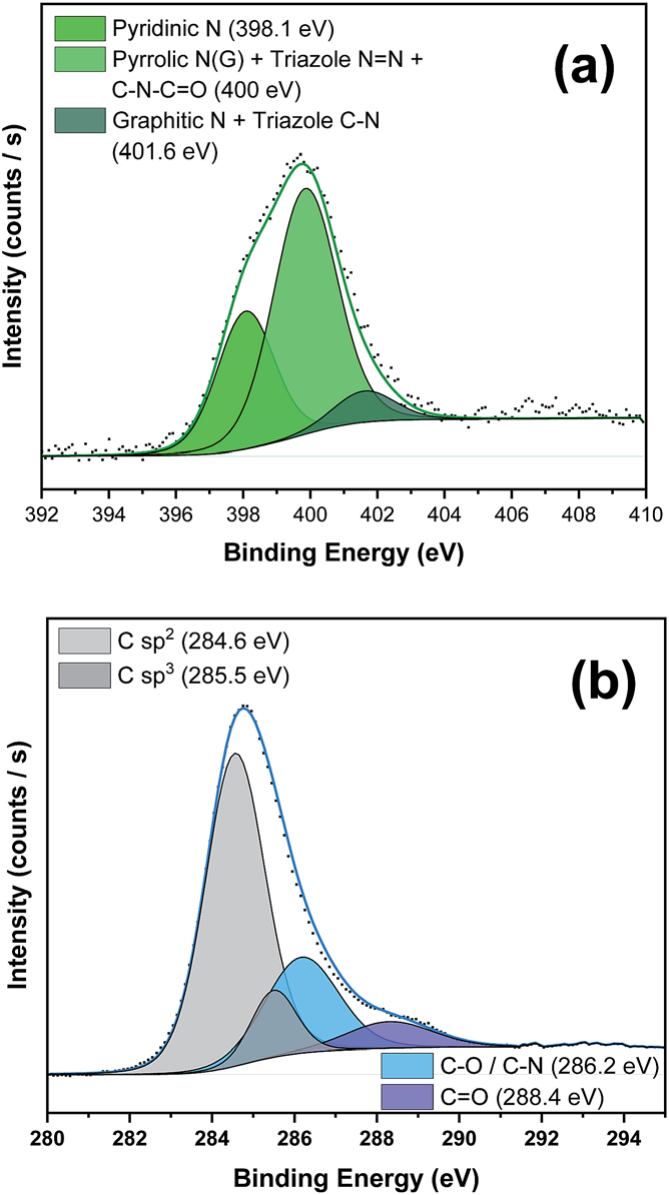
(a) N 1s and (b) C 1s core level XPS regions of NG–PDI 1 and their relative fits.

To further corroborate the presence of the PDI moiety and to confirm the covalent functionalization in the NG material, we carried out X-ray photoemission spectroscopy (XPS) (see Fig. 3 and S4–S6 and the Table S1 in the ESI† for further details). The N 1s spectrum of PDI 5 (Fig. S4a†) displayed two strong components at binding energies of 401.5 eV and 405.1 eV associated with the different nitrogen atoms in the azide group besides the peak at 400.8 eV, corresponding to the nitrogen atom in the imido group.^17^ After the “*click*” reaction (Fig. 3a), the absence of the peak in nanohybrid 1 corresponding to the free azide group at 405 eV unequivocally confirmed the formation of the triazole ring.^18^ Additionally, in the C 1s high-resolution spectrum the presence of sp^3^ C-atoms (285.5 eV) of the alkyl chains of the PDI moiety was detected (Fig. 3b). All these data in concordance with FTIR results clearly support the covalent attachment of 5 to the NG material.

**Fig. 4:**
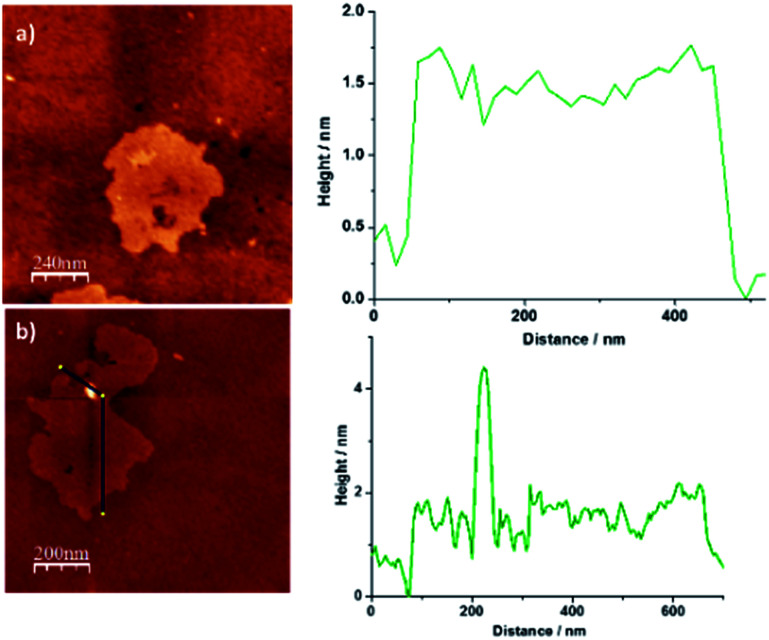
2D AFM images of (a) pristine NG and (b) hybrid material 1 and height profile analysis showing an increase of ∼2 nm.

Finally, additional structural data for NG–PDI 1 were gathered by AFM investigations (Fig. 4). The images obtained for the starting material NG revealed the presence of a few layers of graphene flakes with an average height of *ca.* 2 nm (Fig. 4a). After functionalization, this material showed the existence of brightened zones whose height coincides with the distance calculated for the PDI unit (Fig. S7†) which could suggest the presence of the perylenediimide attached to NG (Fig. 4b).

**Fig. 5:**
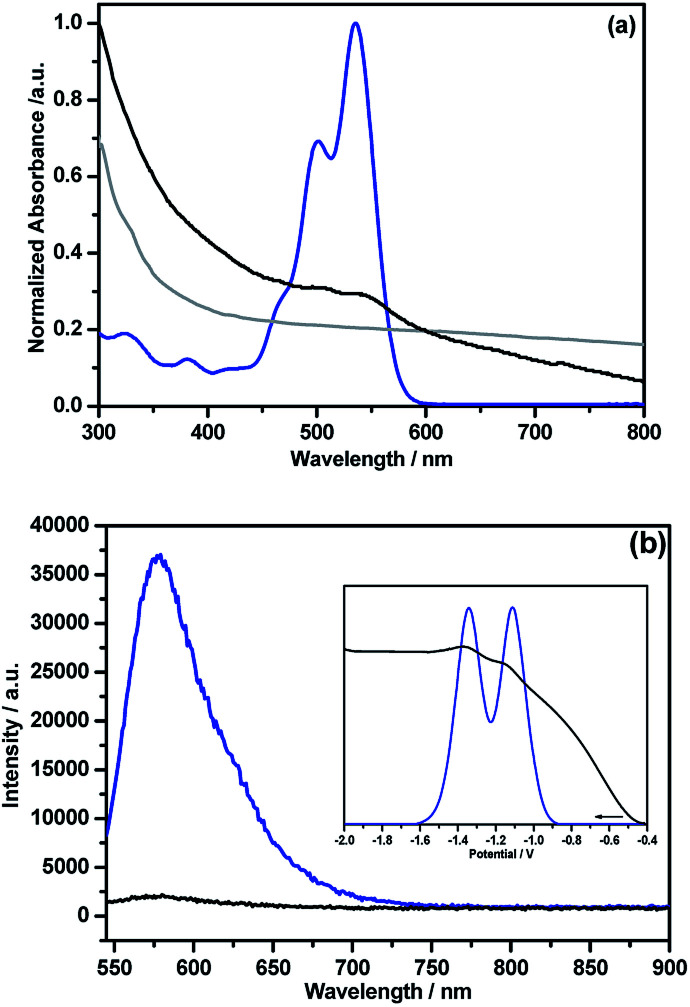
(a) Absorption and (b) fluorescence (*λ*_exc_ = 535 nm) spectra of NG (grey), NG–PDI 1 (black) and PDI 5 (blue) in DCB. (b) Inset shows DPVs of 5 (blue) and 1 (black) in DCB/acetonitrile containing 0.1 M (TBA)ClO_4_.

Unlike pristine NG, the NG–PDI 1 hybrid was found to be soluble in common organic solvents due to the presence of appreciable PDI content. Fig. 5a shows the absorption spectra of the hybrid and control compounds in *o*-dichlorobenzene (DCB). Peaks of PDI in 5 were located at 324, 380, 502 and 535 nm while in 1, these peaks appeared as shoulder peaks to the broad peak of NG with 3–5 nm blue-shift. Such shifts suggests weak intramolecular interactions between the PDI and NG entities.^13^ The fluorescence spectrum of 5, excited at 535 nm corresponding to PDI, revealed a single peak centered at 578 nm; however, in 1 this peak was quenched over 95% of its intensity revealing the occurrence of excited state events in the hybrid (Fig. 5b). The fluorescence lifetime of 5 determined from the time correlated single photon counting technique (TCSPC) and nanoLED excitation source (494 nm) was found to be about 6.7 ns (monoexponential decay, *χ*^2^ = 0.99); however, for 1 it was within the time resolution of our instrumental setup, and hence no lifetime could be measured. As shown in the Fig. 5b inset, the differential pulse voltammogram (DPV) of 5 revealed two reductions at −1.11 and −1.34 V *vs.* Ag/AgNO_3_ in DCB/acetonitrile containing 0.1 M (TBA)ClO_4_. In the hybrid, both reductions experienced a cathodic shift and appeared at −1.15 and −1.37 V, likely due to the presence of intramolecular type interactions between the entities. In the cathodic window, no signals were detected for these materials.

Spectroelectrochemical studies were also performed on 5 to characterize the one-electron reduced product. As shown in Fig. S8,† the electrochemically generated 5˙^−^ revealed a broad peak spanning 620–780 nm with a peak maximum at 724 nm, with additional peaks at 810, 867 and 970 nm. The facile reduction of PDI in the hybrid suggests that it could be an electron acceptor when connected to the N-doped graphene, similar to when this acceptor was connected to strong electron donors such as phthalocyanine and porphyrins.^19–23^ In this donor–acceptor hybrid, upon selective excitation of PDI to its ^1^PDI* state, electron migration from the conduction band of NG to the half-filled HOMO of ^1^PDI* could occur to produce the PDI˙^−^–NG˙^+^ charge separated state.^24^ If such a process occurs, then one would expect to see transient peaks at locations corresponding to PDI˙^−^ of the hybrid. In order to verify this prediction, fs-TA studies were performed and the results are summarized below.

Fig. S9† shows fs-TA spectra in the visible and near-IR regions and at the indicated delay times for 5, NG and the NG–PDI hybrid 1 in DMF. The instantaneously formed ^1^PDI* revealed negative peaks at 501 and 534 nm due to ground state bleaching (Fig. S9a†) and at 618 nm due to stimulated emission.^25^ During the initial time, the 618 nm peak revealed about 10 nm red-shift likely due to vibrational cooling. A broad positive peak centered around 738 nm due to transitions originating from the ^1^PDI* state was also observed. In the near-IR region, 5 also revealed a photoresponse with a broad peak in the 820–1050 nm range with a peak maximum at 990 nm (Fig. S9b†). The decay and recovery of the positive and negative peaks were slow consistent with the long lifetime of 5. The fs-TA spectra of NG shown in Fig. S9c and d† were not well defined, however, they were largely similar to the transient features of graphene dispersions in organic solvents.^26,27^ Mainly, instantaneous formation of optical phonon peaks in the 500, 550, 650 and 1100 nm regions was observed. The signal recovery occurred within about 1.5 ps. The fs-TA spectral features of NG–PDI were appreciably different from those of pristine NG or PDI controls as shown in Fig. S9e and f.† In the visible region, rapid recovery of the ground state bleach and stimulated emission peaks was accompanied by enhanced peak intensities in the 600–700 and 750–800 nm regions. In the near-IR region, the optical phonon peaks of NG (by direct excitation) were broad with a red-shift. A new peak around the 965 nm region was also observed. The new peak positions were supportive of the PDI˙^−^–NG˙^+^ charge separated state. To further establish this, careful analysis and comparison of spectral data were performed, as discussed below.

**Fig. 6:**
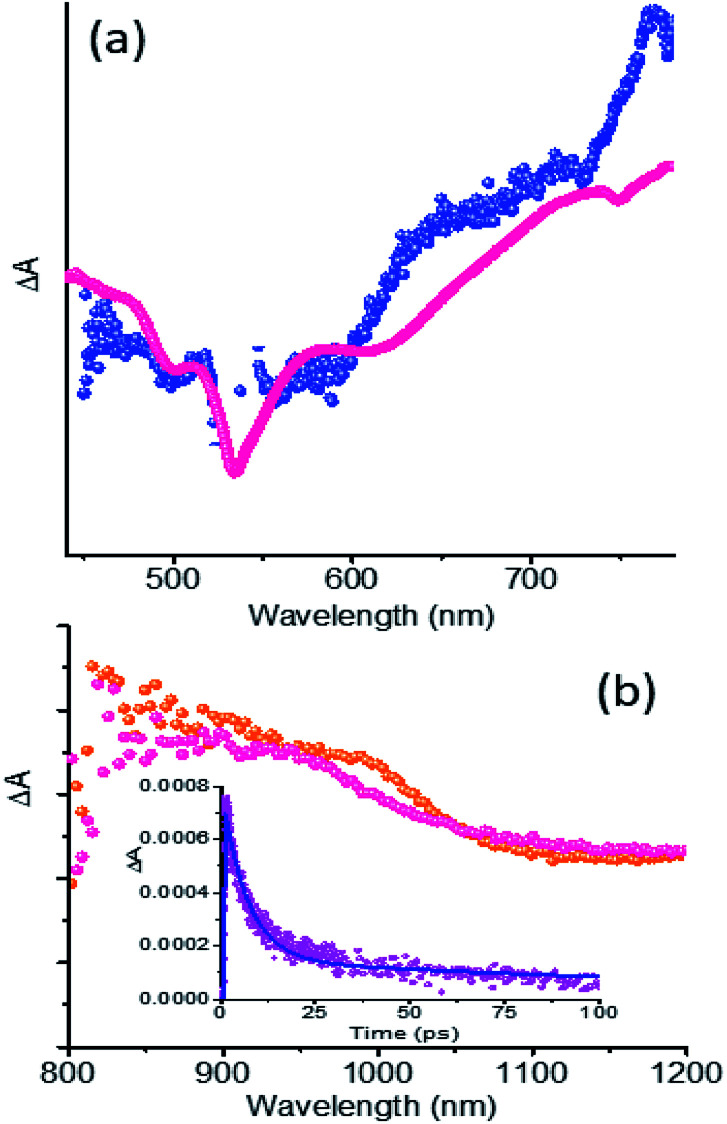
(a) fs-TA spectra at a delay time of 1.5 ps of 5 (magenta) and 1 (blue). (b) fs-TA spectra of 5 (orange) at a delay time of 9 ps and 1 (magenta) at a delay time of 1.7 ps. All spectra were recorded in DMF at the excitation wavelength of 535 nm corresponding to the main absorption of PDI of the hybrid. The (b) inset shows the kinetic profile of the 965 nm peak corresponding to PDI˙^−^. Note: the intensities are not drawn to scale.

The fs-TA spectra of 5 and 1 at 1.5 ps are compared in Fig. 6a. Clear enhancement of absorption signals for the hybrid in the 620–720 nm range and 730–790 nm range corresponding to PDI˙^−^ was obvious. This suggests the overlap of ^1^PDI* originated peaks with those of 5˙^−^ in the spectral range. Similar observations were also made in the near-IR region as shown in Fig. 6b where the fs-TA spectra of 5 at 9 ps and 1 at 1.7 ps are depicted. A good match between the peak of 5˙^−^ from spectroelectrochemical results (Fig. S8†) and the transient peak of the PDI˙^−^–NG˙^+^ charge separated state, sufficiently isolated from the ^1^PDI* signal, was observed. These results clearly demonstrate the occurrence of charge separation from ^1^PDI* to produce the PDI˙^−^–NG˙^+^ charge separated state in the hybrid. In order to secure kinetic information, the growth and decay of the PDI˙^−^ near-IR peak were monitored. Such analysis showed a rate of charge separation of 1.5 × 10^11^ s^−1^ and the rate of charge recombination was 1.4 × 10^9^ s^−1^ (see the Fig. 6b inset for the kinetic profile), suggesting charge stabilization to some extent in this hybrid.

## Conclusions

In summary, for the first time, by using “*click*” chemistry, we have been able to synthesize a donor–acceptor hybrid comprised of nitrogen doped graphene with a well-known photosensitizer PDI that acts as an electron acceptor (NG–PDI 1). The TGA and XPS results revealed the incorporation of the PDI moiety into 1 due to the adopted “*click*” chemistry. Spectral studies involving FT-IR and UV-vis techniques indicated the existence of ground state interactions that were also confirmed by electrochemical studies. The occurrence of excited state events was indicated by both steady-state and time-resolved emission studies where significant quenching of PDI fluorescence by NG was observed. Finally, fs-TA studies provided evidence of charge separation from ^1^PDI* to produce the PDI˙^−^–NG˙^+^ charge separated state as the product. Charge stabilization to some extent was also observed in the hybrid suggesting that this novel material could be useful for designing a new generation of energy harvesting and fuel producing solar devices.

## Conflicts of interest

There are no conflicts to declare.

## Supplementary Material

NA-001-C9NA00416E-s001
