# Supplementary material for: Occurrence of excited state charge separation in a N-doped graphene–perylenediimide hybrid formed via ‘click’ chemistry
Source: Nanoscale Adv. 2019 Aug 30;1(10):4009–15. doi: 10.1039/c9na00416e (PMC9418608; doi:10.1039/c9na00416e)
Supplement: NA-001-C9NA00416E-s001 [file NA-001-C9NA00416E-s001.pdf]

Electronic Supplementary Material (ESI) for Nanoscale Advances.
This journal is © The Royal Society of Chemistry 2019

Supporting Information

Occurrence of Excited State Charge Separation in *N*-Doped Graphene-Perylenediimide Hybrid Formed via 'Click' Chemistry

Habtom B. Gobeze, † Luis M. Arellano‡, Ana María Gutiérrez-Vílchez§, María J. Gómez-Escalonilla,‡ Ángela Sastre-Santos,§ Fernando Fernández-Lázaro,§* Fernando Langa‡* and Francis D'Souza†*

†Department of Chemistry, University of North Texas, 1155 Union Circle, #305070, Denton, TX 76203-5017

‡ Universidad de Castilla-La Mancha, Instituto de Nanociencia, Nanotecnología y Materiales Moleculares (INAMOL),45071-Toledo, Spain

§Área de Química Orgánica, Instituto de Bioingeniería, Universidad Miguel Hernández, Avda. de la Universidad, s/n, Elche 03202, Spain

CONTENTS

1. Supplementary Figures and Tables

Figure S1. TGA profiles of NG samples and PDI **5**......**S3**

Figure S2. Raman spectra of NG-TMS derivative...... **S4**

Figure S3. FTIR spectra of NG samples and PDI **5**......**S5**

Figure S4: N1s and C1s core-level spectra of **5**......**S6**

Figure S5: N1s and C1s core-level spectra of **NG**......**S7**

Figure S6: N1s, C1s and Si 2p core-level spectra of **NG-TMS**......**S8**

Figure S7: Molecular mechanic calculations of **1**......**S9**

Figure S8: Absorption spectrum of **5**......**S10**

Figure S9: Femtosecond transient absorption spectra of **5** and **NG-PDI 1**......**S11**

Table S1. XPS atomic percentages (at.%) of *functionalized* **NG samples** and **NG** and **5** precursors...... **S12**

2. Synthetic details and characterization...... **S13**

Figure S10: ^{1}H-NMR spectrum of **3** in $CDCl_3$......**S15**

Figure S11: ^{13}C-NMR spectrum of **3** in $CDCl_3$......**S15**

Figure S12: HR-MALDI-TOF spectrum of **3**......**S16**

Figure S13: ^{1}H-NMR spectrum of **4** in $CDCl_3$ **S17**

Figure S14: ^{13}C-NMR spectrum of **4** in $CDCl_3$ **S17**

Figure S15: HR-MALDI-TOF spectrum of **4** **S18**

Figure S16: ^{1}H-NMR spectrum of **5** in $CDCl_3$ **S19**

Figure S17: ^{13}C-NMR spectrum of **5** in $CDCl_3$ **S19**

Figure S18: HR-MALDI-TOF spectrum of **5** **S20**

References.. **S21**

1. Supplementary Figures and Tables

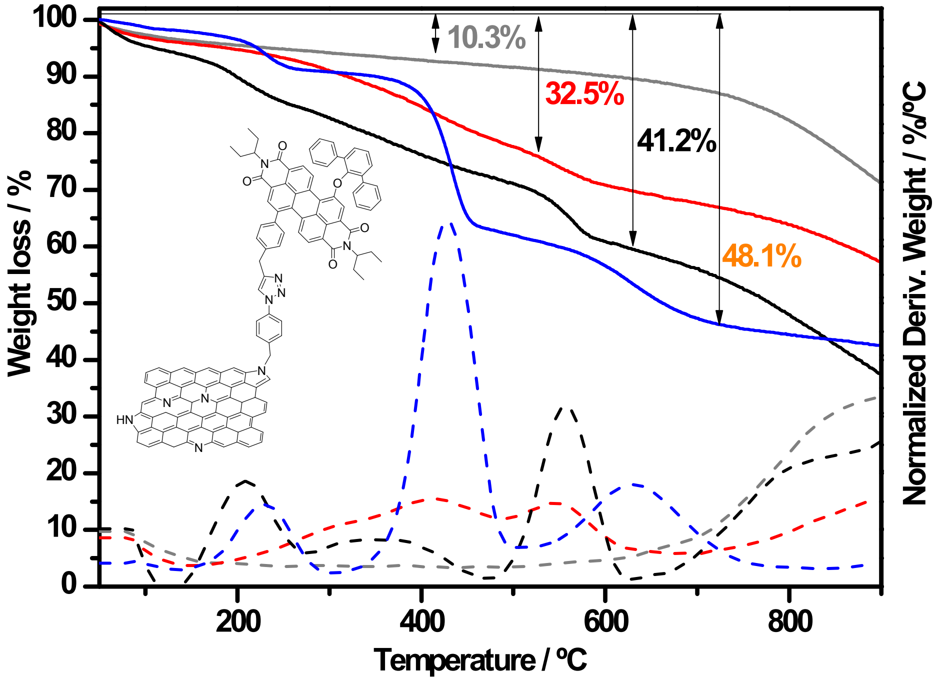

Figure S1. TGA profiles of **NG** (—), **NG-TMS** (—), PDI **5** (—), NG-PDI **1** (—) along with their respective first derivatives (dashed lines) obtained under a nitrogen atmosphere.

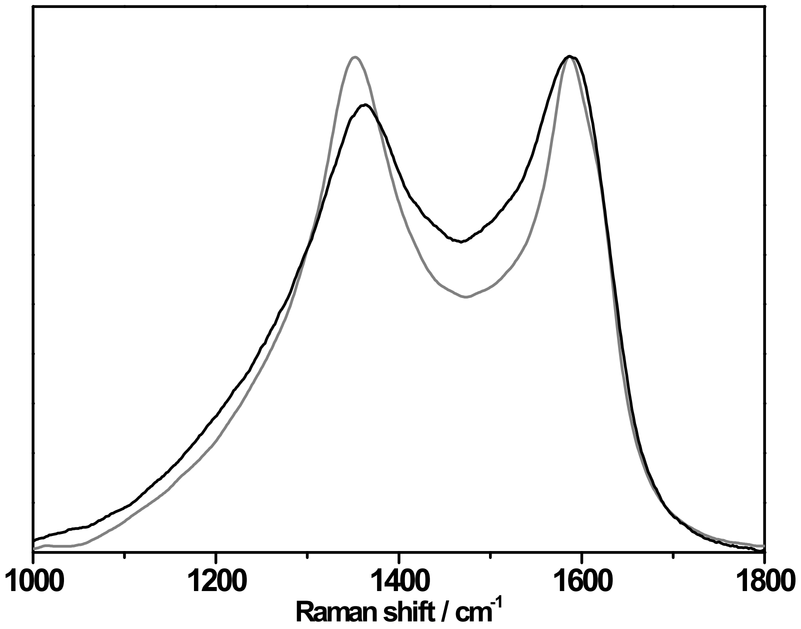

Figure S2. Details of the G-band Raman region (from 1000 to 1800 cm^{-1}) for NG (—) compared with **NG-TMS** derivative (**—**).

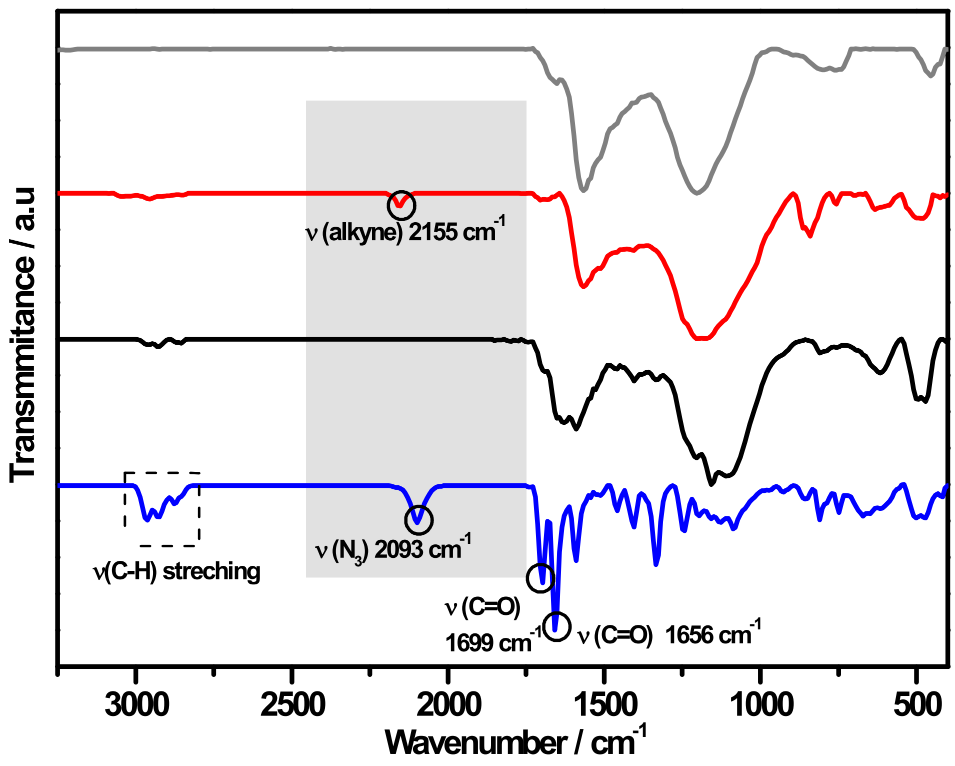

Figure S3. FTIR spectra of **NG-PDI 1** (—) compared with the corresponding spectra of **NG** (—), **NG-TMS** (—) and PDI **5** (—)**.** Coloured area highlight typical region for ν(N_3) and ν(alkyne) vibrations.

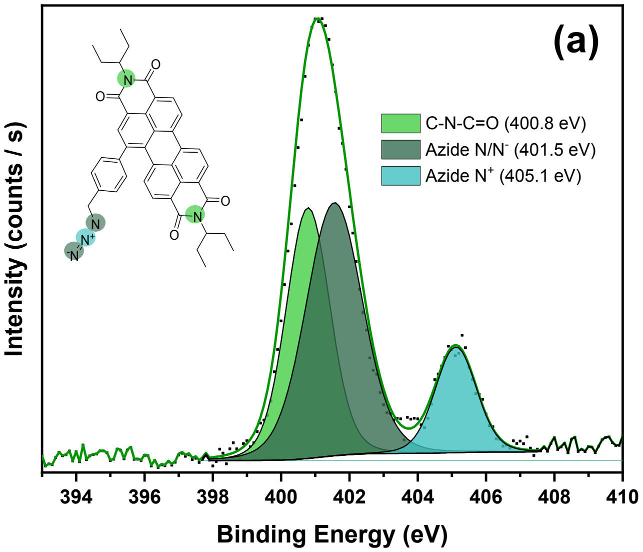

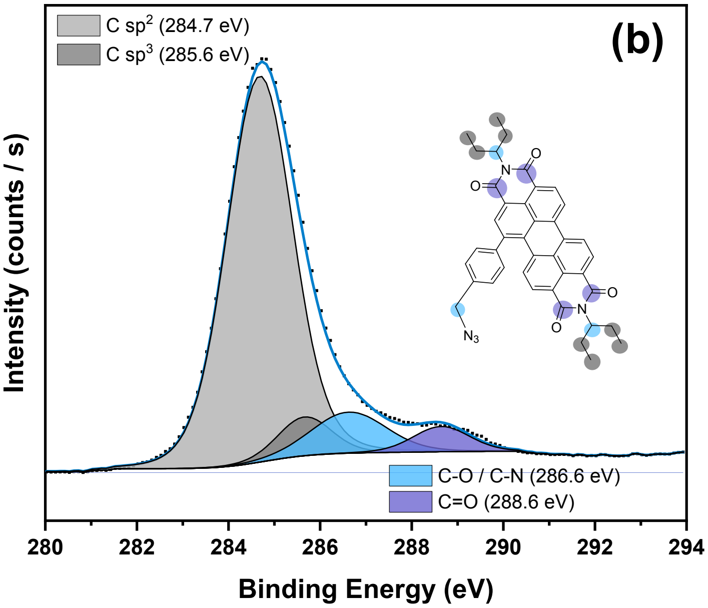

C 1s	BE (eV)	at. %
**C sp^2**	284.7	80
**C sp^3**	285.6	6
**C-O / C-N**	286.6	10

N 1s	BE (eV)	at. %
**C-N-C=O**	400.8	40
**Azide N/N$^-$**	401.5	41
**Azide N$^+$**	405.1	19

Figure S4. N1s (a) and C1s (b) core level regions of **5** and their deconvolutions.

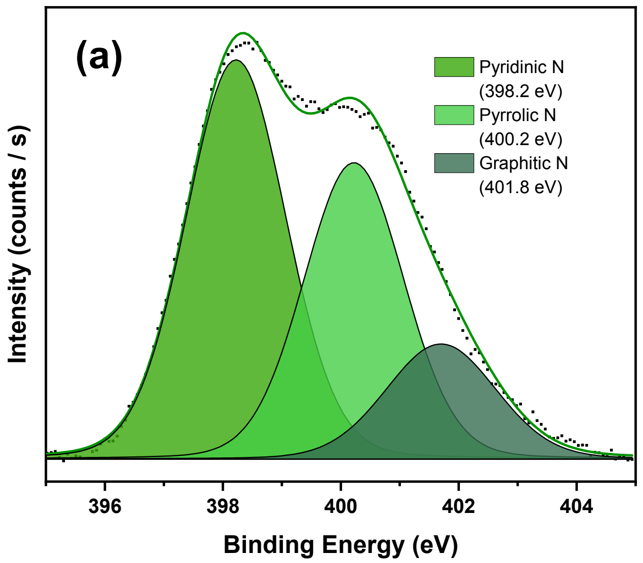

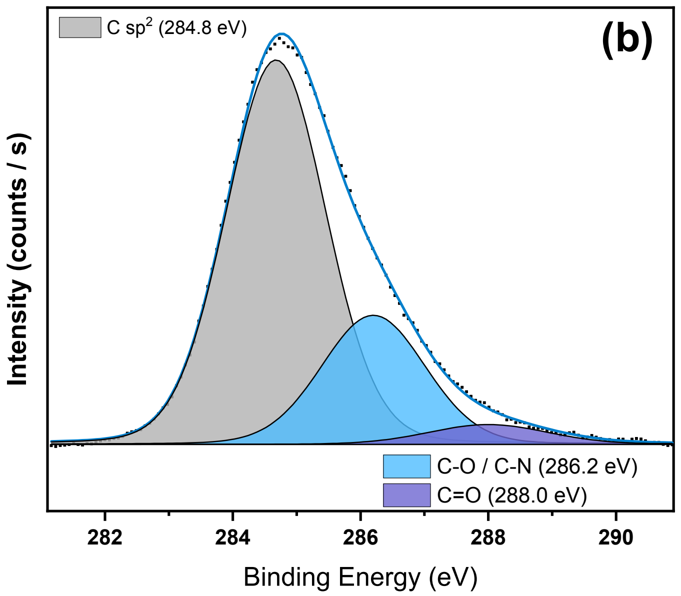

C 1s	**BE (eV)**	**at. %**
**C sp^2**	284.8	72
**C-O / C-N**	286.2	23
**C=O**	288.0	5

N 1s	**BE (eV)**	**at. %**
**Pyridinic N**	398.2	49
**Pyrrolic N**	400.2	36
**Graphitic N**	401.8	15

Figure S5 . N1s (a) and C1s (b) core level regions of starting material **NG** and their deconvolutions.

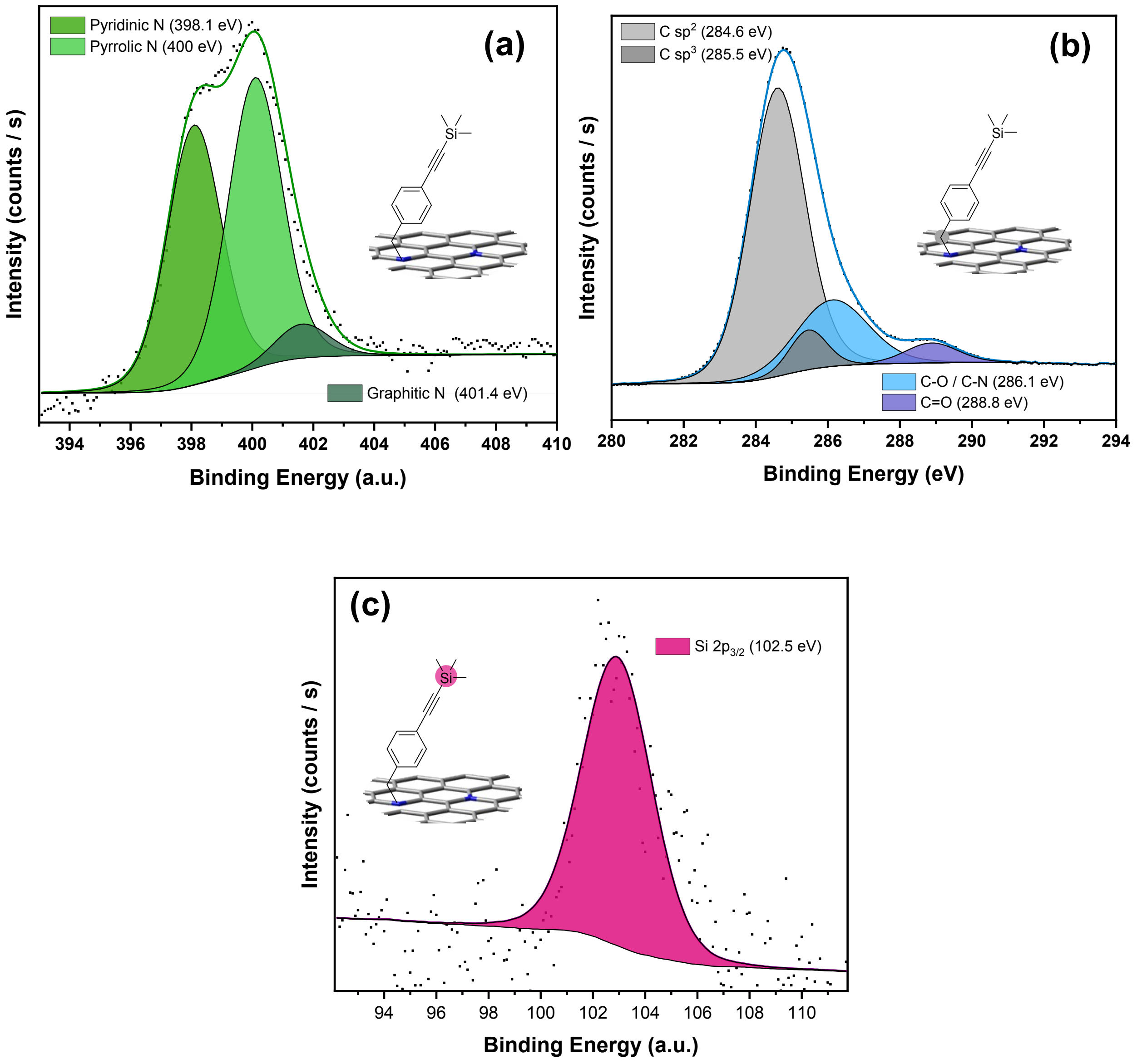

C 1s	BE (eV)	at. %
C sp^2	284.6	70
C sp^3	285.5	6
C-O / C-N	286.1	20
C=O	288.8	4

N 1s	BE (eV)	at. %
Pyridinic N	398.1	43
Pyrrolic N	400	51
Graphitic N	**401.4**	6

Si 2p$_{3/2}$	BE (eV)	at. %
Si	102.5	100

Figure S6. N1s (a), C1s (b) and Si 2p core level regions of **NG-TMS** and their deconvolutions.

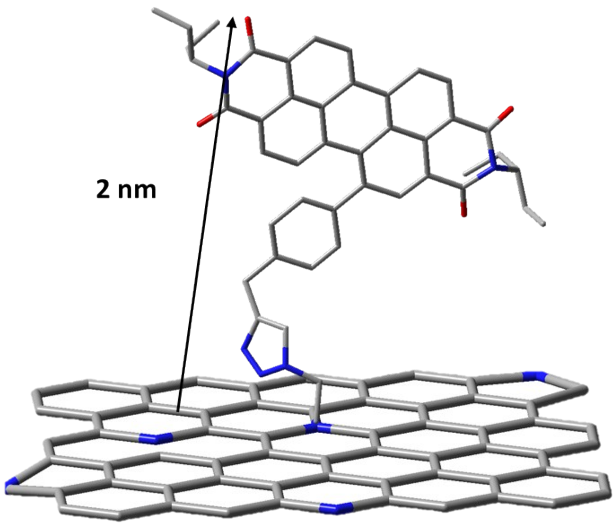

Figure S7. Modelling structure optimized using semiempirical PM3 method implemented on HyperChem 8.0 program package for NG-PDI **1**.

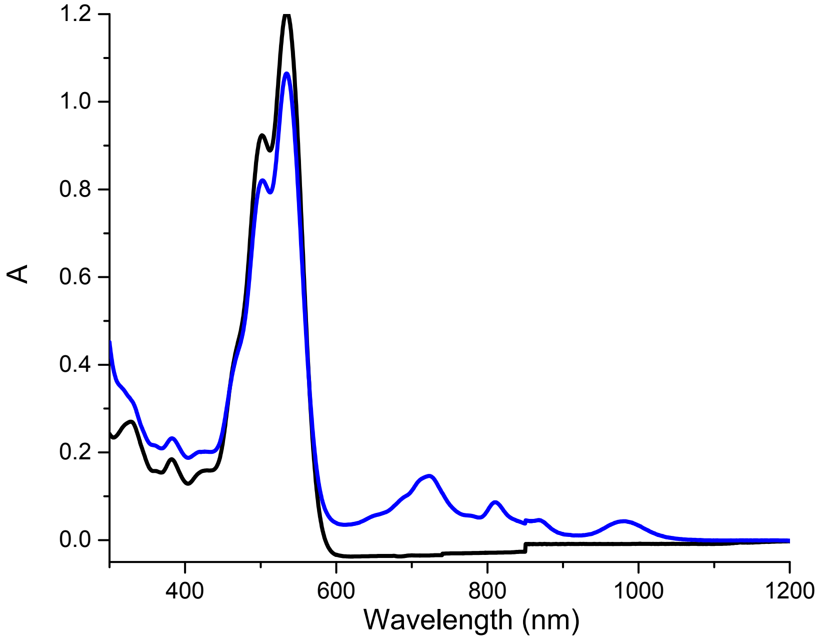

Figure S8. Absorption spectrum of **5** (—) and **5**$^{\cdot-}$ (—) generated by bulk electrolysis at an applied voltage of -0.9 V in DMF containing 0.2 M (TBA)ClO_4.

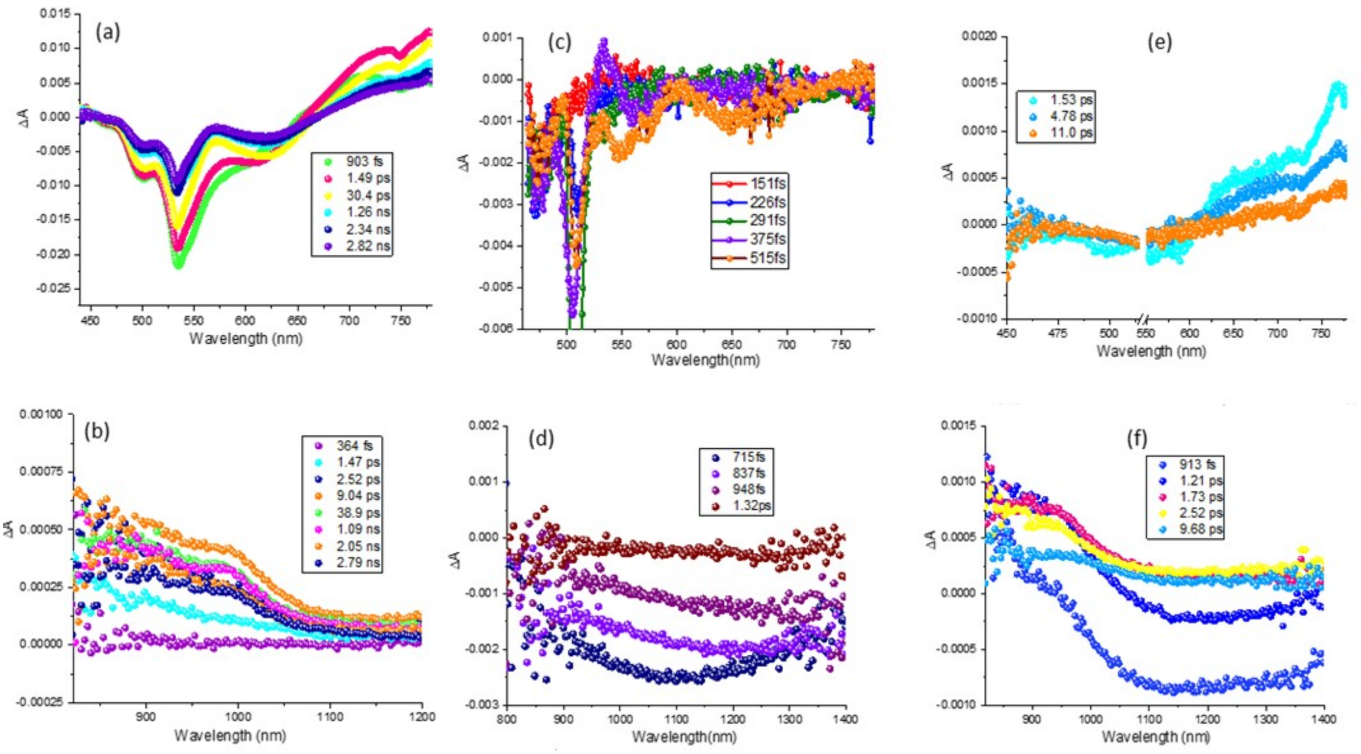

Figure S9. Femtosecond transient absorption spectra at the indicated delay times of (a and b) **5**, (c and d) **NG**, and (e and f) **1** in degassed DMF. **5** and **1** were excited at 535 nm corresponding to PDI excitation while **NG** was excited at 440 nm.

Table S1. Composition of atomic ratios of NG functionalized samples and precursors **NG** and **5**, determined from the XPS survey spectra.

Sample	Core	Atomic (%)
**5**	C1s	86.5
	N1s	2.6
	O1s	10.9
**NG**	C1s	95.5
	N1s	2.8
	O1s	1.7*
**NG-TMS**	C1s	88.9
	N1s	2.7
	O1s	6.9**
	Si2p	1.5
**NG-PDI 1**	C1s	83.1
	N1s	3.1
	O1s	13.8

***** The O1s peak is observed in NG sample, which is possibly due to physisorbed oxygen on the graphene surface, due to the good oxygen adsorption ability in this material.[2]

** Due to the ultrasonication process in liquid exfoliation, the oxidative processes increase, resulting in a higher content of oxidized carbon atoms.[3]

2. Synthetic details and characterization

Synthesis of *N,N'*-di(1'-ethylpropyl)-1-(4''-hydroxymethylphenyl)perylene-3,4:9,10-tetracarboxydiimide (3). A solution of Na_2CO_3 in Milli-Q water (17.9 mg in 1.55 mL) is added to a mixture of **2**[1] (100 mg, 0.16 mmol), 4-(hydroxymethyl)phenylboronic acid (28 mg, 0.19 mmol), $Pd(PPH_3)_4$ (18 mg, 0.016 mmol) and dry THF (13 mL). This solution is stirred 12 h at 70 ºC under N_2. After cooling at room temperature some dichloromethane is added and the solution is dried with sodium sulfate. After the solvent is distilled off the reaction crude is purified by column chromatography (SiO_2, chloroform/acetone: 30/1) to afford **3** (89 mg, 87%) as a dark red solid. ^{1}H NMR (300 MHz, $CDCl_3$, 25 ºC): 8.69-8.55 (5H, m, 5xPDI-*H*), 8.11 (1H, d, *J*=8.2 Hz, 1xPDI-*H*), 7.86 (1H, d, *J*=8.2 Hz, 1xPDI-*H*), 7,55-7.26 (4H,m, 4xAr-*H*), 5.11-4.97 (2H, m, 2xPDI-C*H*$(CH_2CH_3)_2$), 4.85 (2H, s, Ar-C*H*$_2$-OH), 2.34-2.15 (4H, m, 2xPDI-CH(C*H*H$CH_3)_2$), 2.02-1.84 (4H, m, 4xPDI-CH(CH*H*$CH_3)_2$),1.63 (1H, brs, -O*H*), 0.93 (6H, t, *J*=7.5 Hz, 1xPDI-CH(CH_2C*H*$_3)_2$) and 0.87 (6H, t, *J*=7.5 Hz, 1xPDI-CH(CH_2C*H*$_3)_2$) ppm; ^{13}C NMR (75 MHz, $CDCl_3$, 25 ºC): δ 141.82, 141.58, 141.45, 134.80, 134.67, 134.41, 132.53, 129.93, 128.71, 128.69, 128.56, 128.07, 127.54, 123.49, 122.64, 64.79, 57.76, 57.64, 25.03, 25.00 and 11.28 ppm; IR-FT (KBr) ν/cm^{-1}: 3449, 2965, 2933, 2876, 1697, 1654, 1590, 1460, 1421, 1406, 1364, 1332, 1247, 1200, 1085, 811, 789, 750; UV/vis (CH_2Cl_2), λ_{max}/nm (logε): 507 (4,60), 536 (4,66); HR-MS (MALDI-TOF, dithranol): *m/z*=636.2695, $[M]^-$, calcd for $C_{41}H_{36}N_2O_5$: 636.2624

Synthesis of *N,N'*-di(1'-ethylpropyl)-1-(4''-bromomethylphenyl)perylene-3,4:9,10-tetracarboxydiimide (4). A solution of **3** (168 mg, 0.264 mmol) in dry CH_2Cl_2 (32 mL) is stirred for a few minutes under nitrogen. Then, PPh_3 (226 mg, 0.861 mmol) is added and, finally, CBr_4 (285 mg, 0.861 mmol) is throwed in. The mixture is heated for 1h at 50ºC. Then, the solution is cooled at room temperature and washed with saturated sodium bicarbonate solution. The resulting organic solution is dried with sodium sulfate. After distilling off the solvent, the reaction crude is purified by column cromatography (SiO_2, dichloromethane) yielding **4** (85.4 g, 46%) as a dark pink solid. ^{1}H NMR (300 MHz, $CDCl_3$, 25 ºC): 8.69-8.54 (5H, m, 5xPDI-*H*), 8.13 (1H, d, *J*=8.2, 1xPDI-*H*), 7.81 (1H, d, *J*=8.2, 1xPDI-*H*), 7.58-7.44 (4H, m, 4xAr*H*), 5.12-4.98 (2H, m, 2xPDI-C*H*$(CH_2CH_3)_2$), 4.60 (2H, s, Ar-C*H*$_2$-Br), 2.35-2.16 (4H, m, 2xPDI-CH(C*H*H$CH_3)_2$), 2.01-1.84 (4H, m, 4xPDI-CH(CH*H*$CH_3)_2$), 0.93 (6H, t, *J*=7.5 Hz, 1xPDI-CH(CH_2C*H*$_3)_2$ and 0.90ppm (6H, t, *J*=7.5 Hz, 1xPDI-CH(CH_2C*H*$_3)_2$ ppm; ^{13}C NMR (75 MHz, $CDCl_3$, 25 ºC): 142.58, 140.85, 140.81, 138.45, 138.03, 134.69, 134.38, 134.25, 132.47, 130.97, 130.47, 129.95, 129.08, 128.95, 128.89, 128.52, 127.95, 127.40, 123.49, 122.66, 57.67, 57.55, 45.56, 32.56, 29.64, 24.95, 24.93 and 11.28 ppm; IR-FT (KBr) ν/cm^{-1}: 3422, 2963, 2932, 2875, 1698, 1655, 1591, 1460, 1406, 1331, 1247, 1201, 1084, 847, 811, 748, 612; UV/vis (CH_2Cl_2), λ_{max}/nm (logε): 503 (4.39), 536 (4.53); HR-MS (MALDI-TOF, dithranol): *m/z*=698.1783, $[M]^-$, calcd for $C_{41}H_{35}BrN_2O_4$: 698.1780.

Synthesis of *N,N'*-di(1'-ethylpropyl)-1-(4''-azidomethylphenyl)perylene-3,4:9,10-tetracarboxydiimide (5). Sodium azide (741.1 mg, 114 mmol) and **4** (79.6 mg, 0.114 mmol) were disolved in DMF-H_2O (10:1, 10 mL) and heated to 70ºC for 4 h. After cooling down to room temperature, the solvent was removed. The reaction crude was purified by column cromatography (SiO_2, dichloromethane) yielding **1** (57.4g, 76%) as a dark pink solid. ^1H NMR (300 MHz, $CDCl_3$, 25 ºC): 8.71-8.56 (5H, m, 5xPDI-*H*), 8.12 (1H, d, *J*=8.2, 1xPDI-*H*), 7.78 (1H, d, *J*=8.2, 1xPDI-*H*), 7.55-7.48 (4H, m, 4xAr*H*), 5.13-4.92 (2H, m, 2xPDI-C*H*(CH_2CH_3)$_2$), 4.49 (2H, s, Ar-CH_2-N_3), 2.35-2.16 (4H, m, 2xPDI-CH(C*H*HCH_3)$_2$), 2.01-1.84 (4H, m, 4xPDI-CH(CH*H*CH_3)$_2$), 0.93 (6H, t, *J*=7.5 Hz, 1xPDI-CH(CH_2C*H*$_3$)$_2$ and 0.87 (6H, t, *J*=7.5 Hz, 1xPDI-CH(CH_2C*H*$_3$)$_2$)ppm; ^{13}C NMR (75 MHz, $CDCl_3$, 25 ºC): 142.59, 140.89, 135.73, 134.78, 134.44, 134.30, 132.58, 130.18, 129.96, 129.13, 129.05, 128.58, 128.00, 127.46, 123.52, 122.70, 64.53, 57.71, 57.58, 54.34, 24.99, 24.96 and 11.30 ppm; IR-FT (KBr) v/cm^{-1}: 2964, 2932, 2875, 2099, 1698, 1657, 1460, 1406, 1331, 1246, 1200, 1161, 1084, 866, 851, 812, 789, 749; UV/vis (CH_2Cl_2), λ_{max}/nm (logε): 505 (4.57), 535 (4.66); HR-MS (MALDI-TOF, dithranol): *m/z*=661,2691, $[M]^-$, calcd for $C_{41}H_{35}N_5O_4$: 661.2689.

Characterization

***N,N'-di(1'-ethylpropyl)-1-(4''-hydroxymethylphenyl)perylene-3,4:9,10-tetracarboxydiimide* (3)**

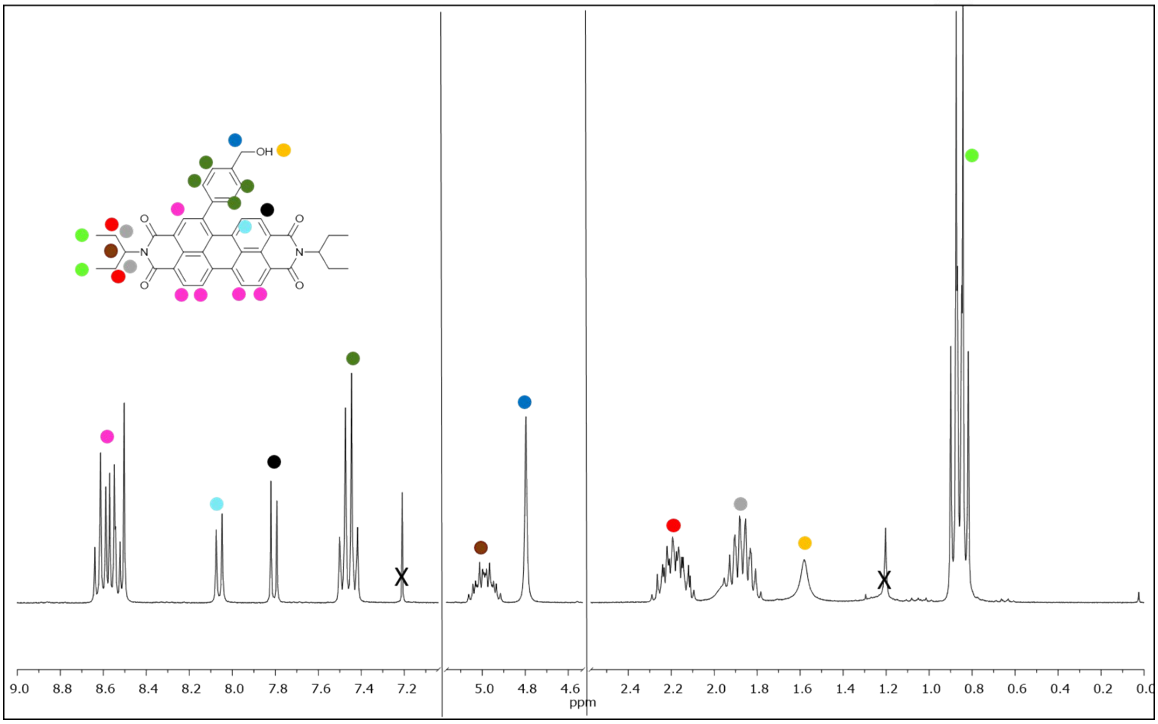

Figure S10: ^{1}H-NMR spectrum of **3** in $CDCl_3$

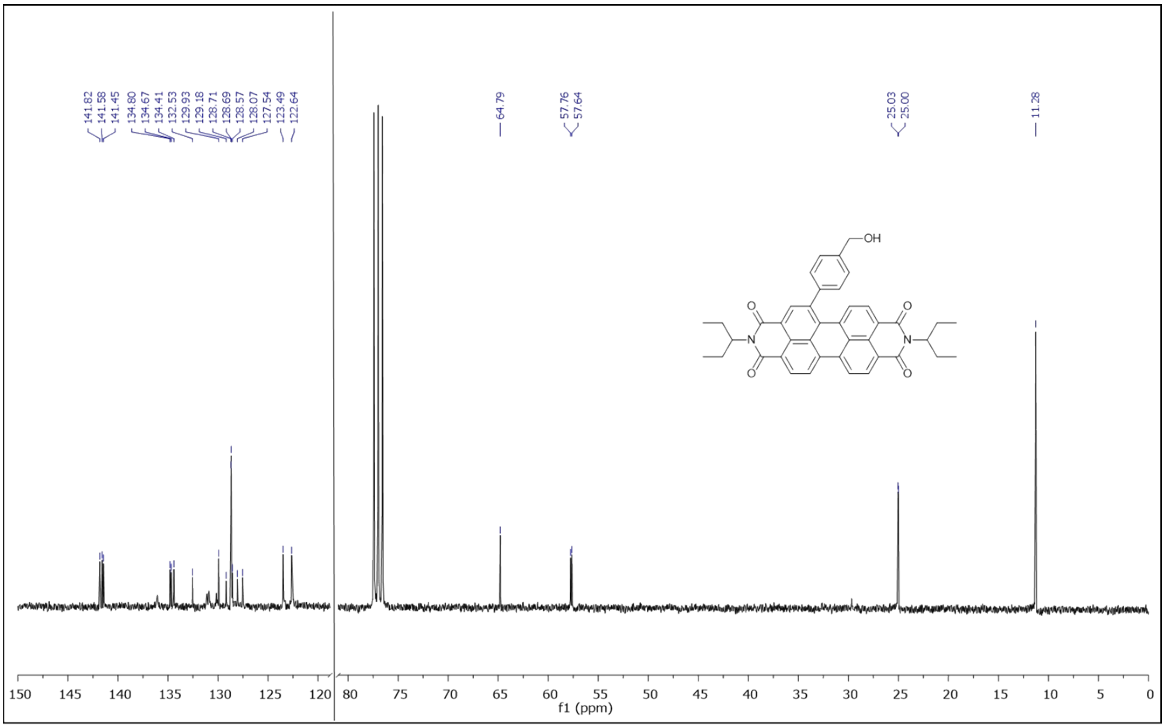

Figure S11: ^{13}C-NMR spectrum of **3** in $CDCl_3$

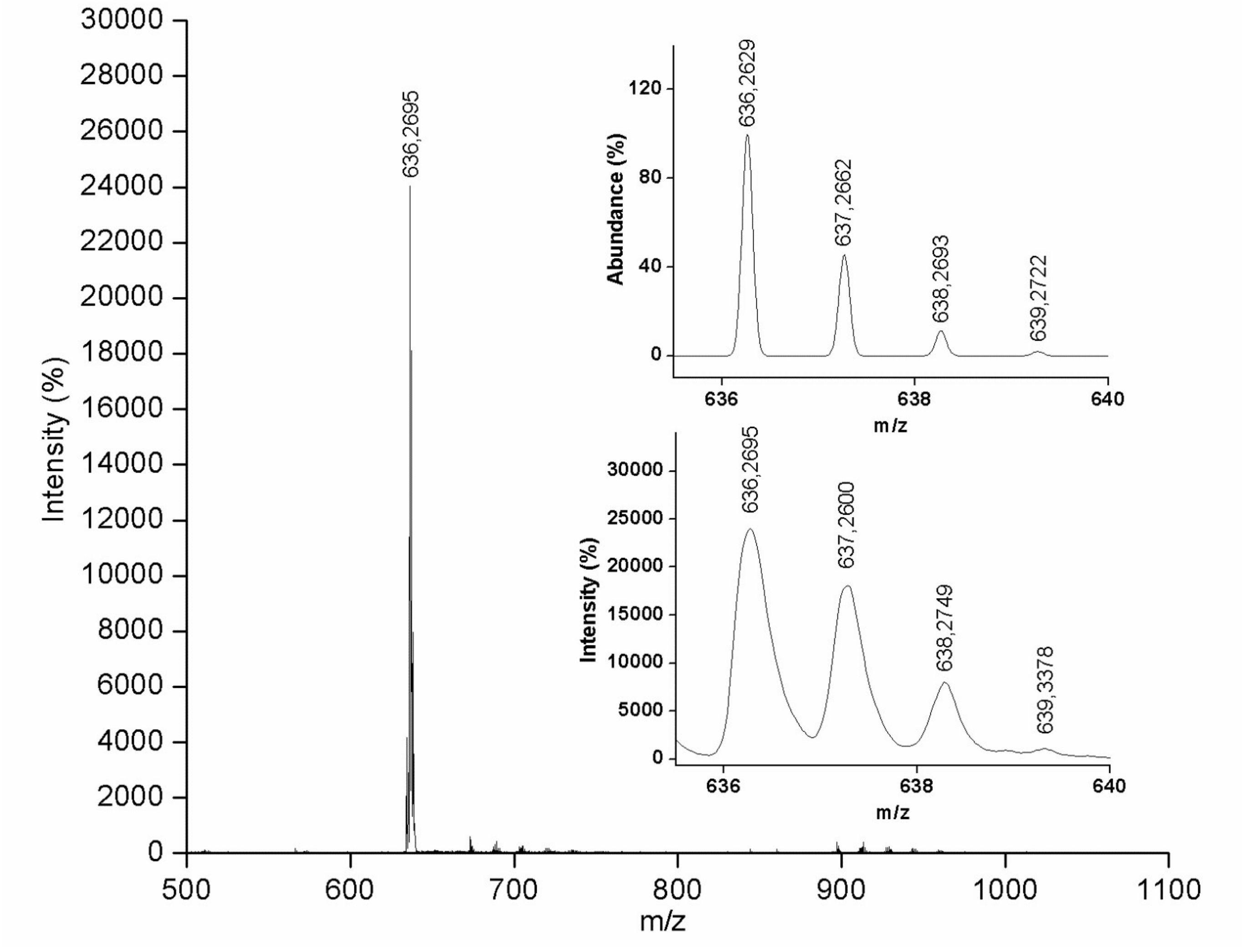

Figure S12: HR-MALDI-TOF spectrum of **3**

N,N'-di(1'-ethylpropyl)-1-(4''-bromomethylphenyl)perylene-3,4:9,10-tetracarboxydiimide (4)

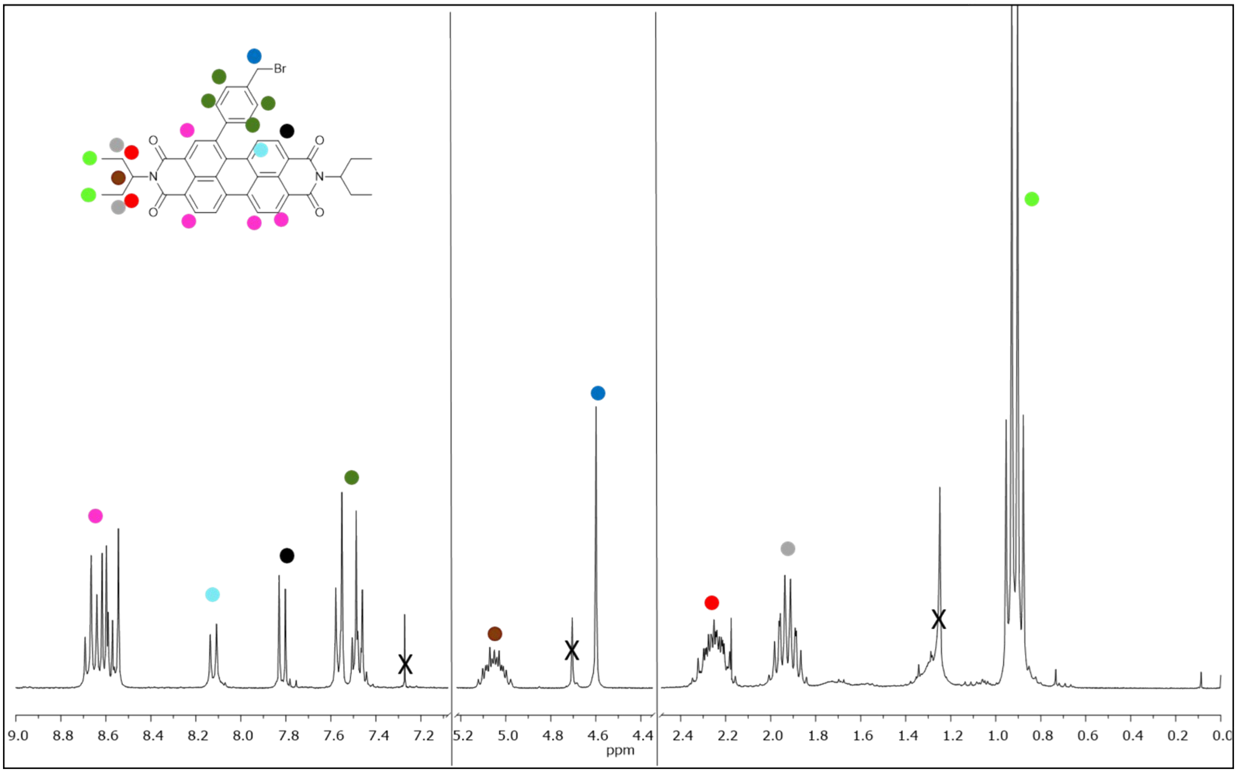

Figure S13: ^{1}H-NMR spectrum of **4** in $CDCl_3$

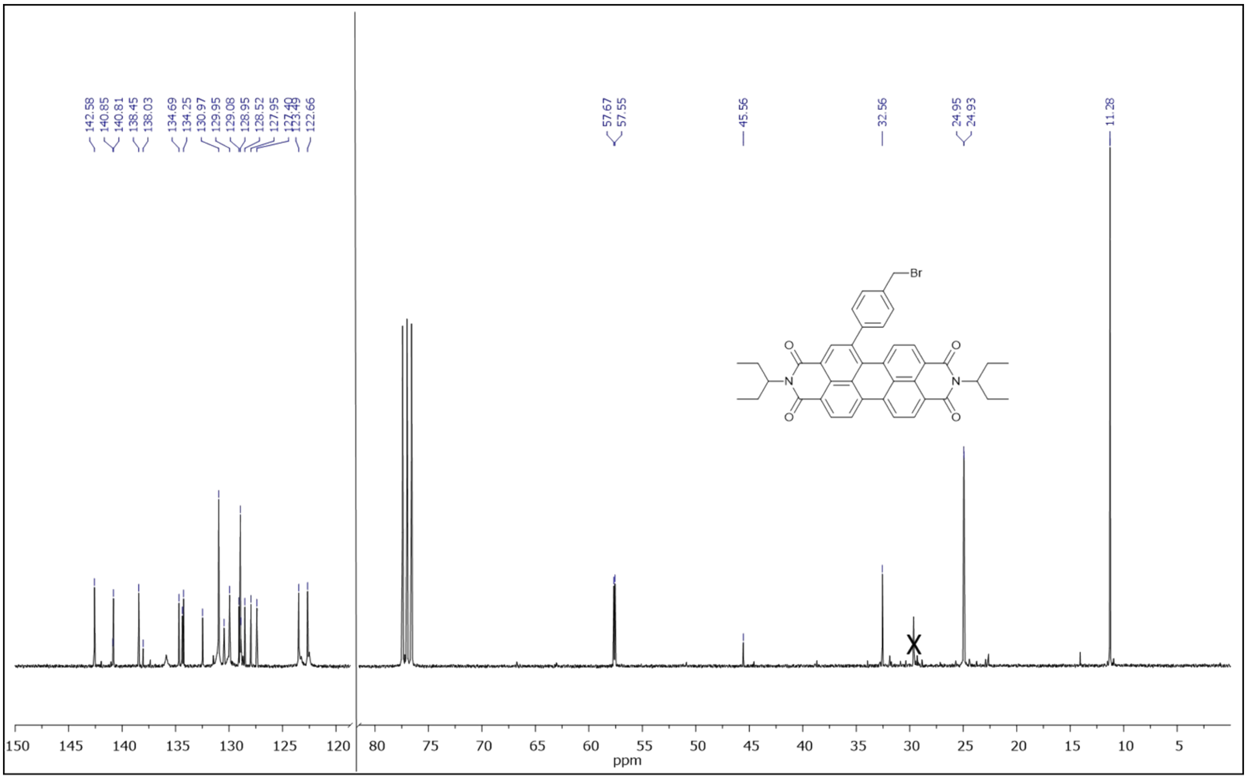

Figure S14: ^{13}C-NMR spectrum of **4** in $CDCl_3$

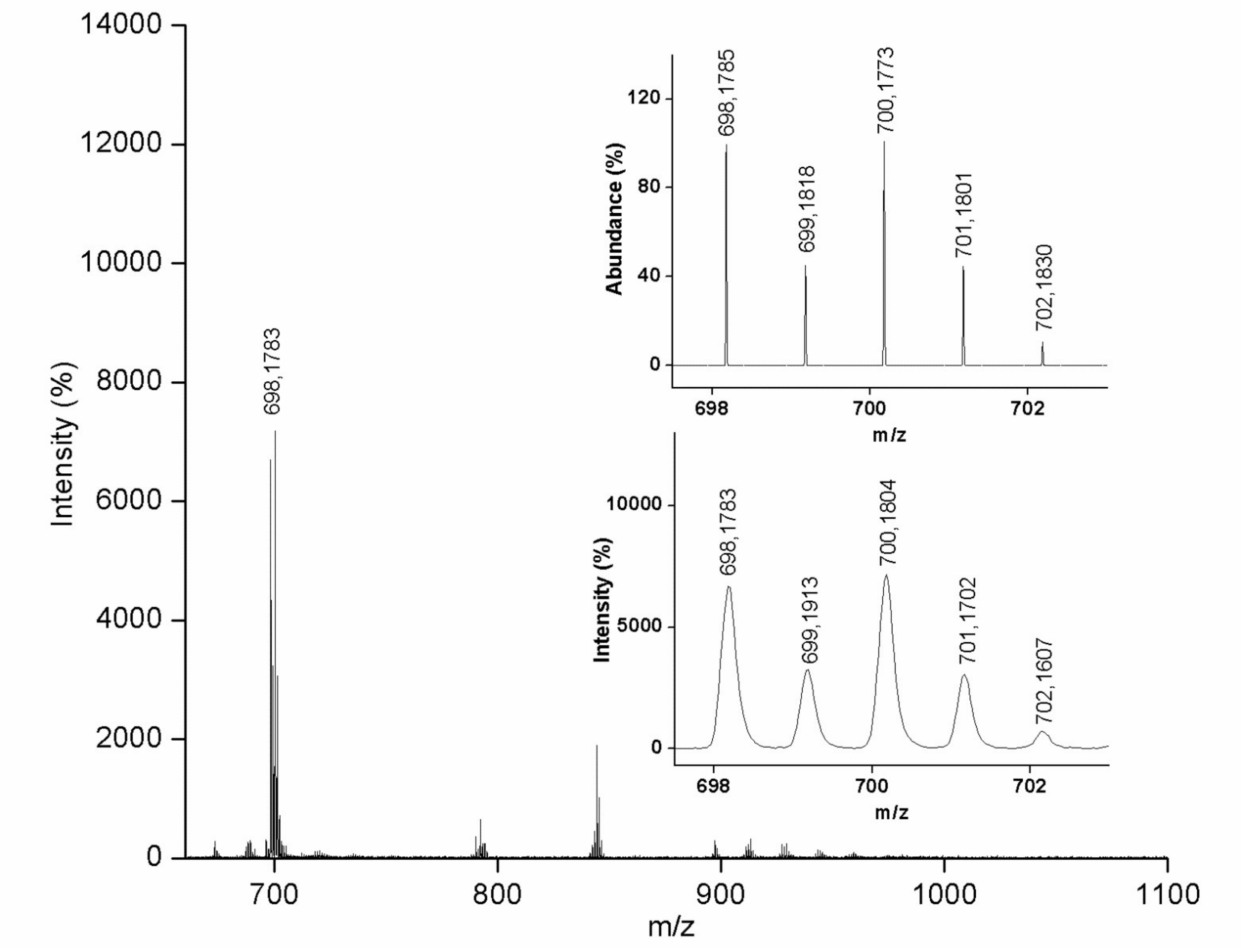

Figure S15: HR-MALDI-TOF spectrum of **4**

N,N’-di(1’-ethylpropyl)-1-(4’’-azidomethylphenyl)perylene-3,4:9,10-tetracarboxydiimide (5)

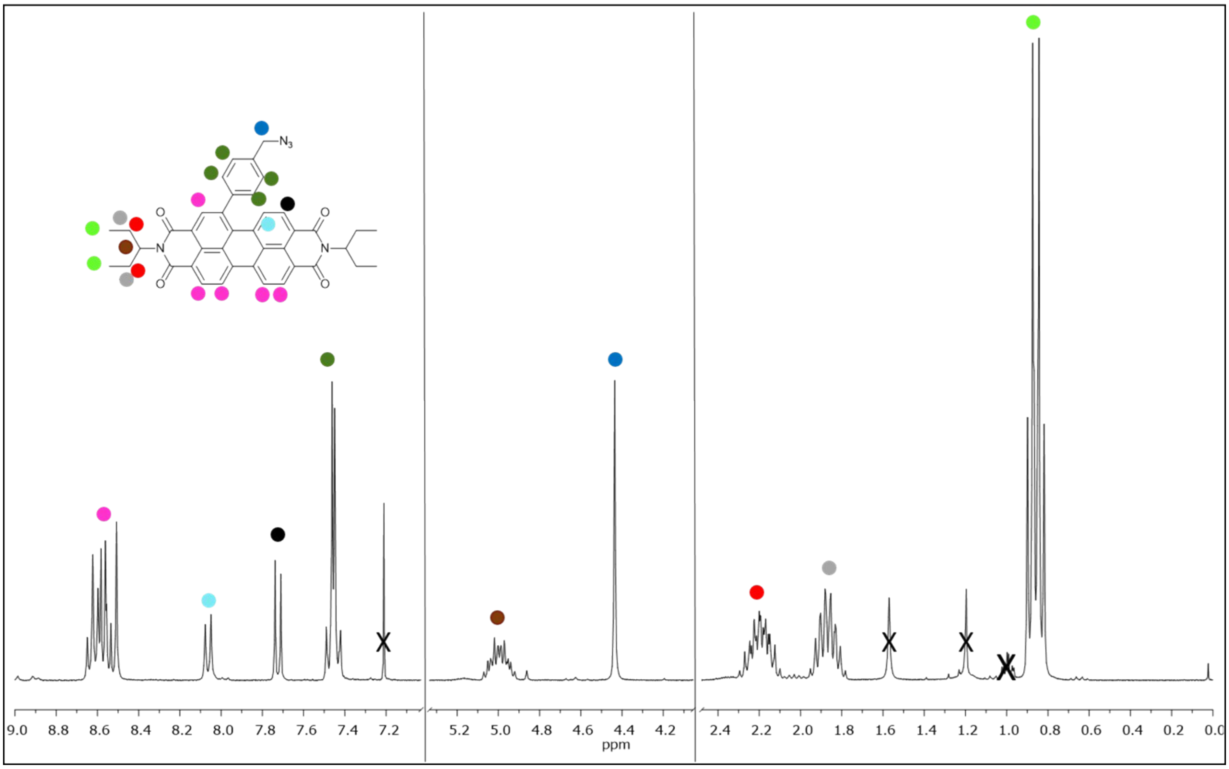

Figure S16: ^{1}H-NMR spectrum of **5** in $CDCl_3$

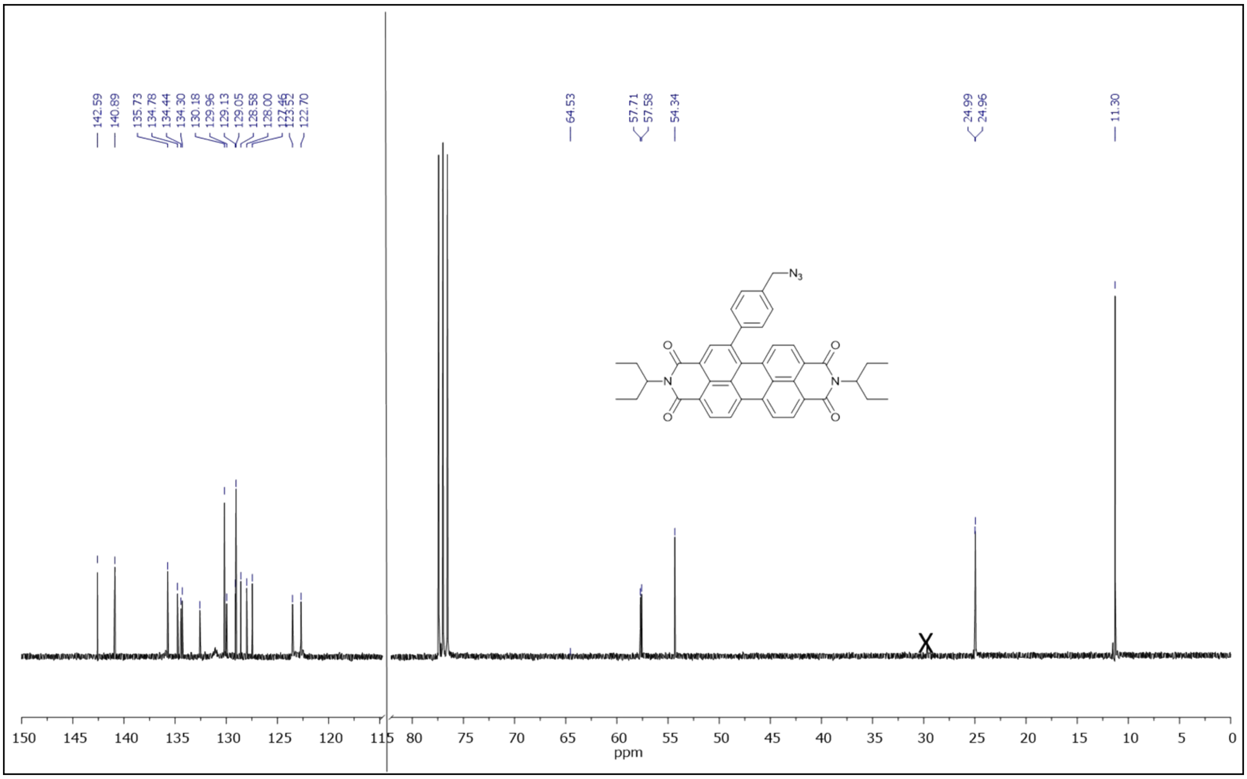

Figure S17: ^{13}C-NMR spectrum of **5** in $CDCl_3$

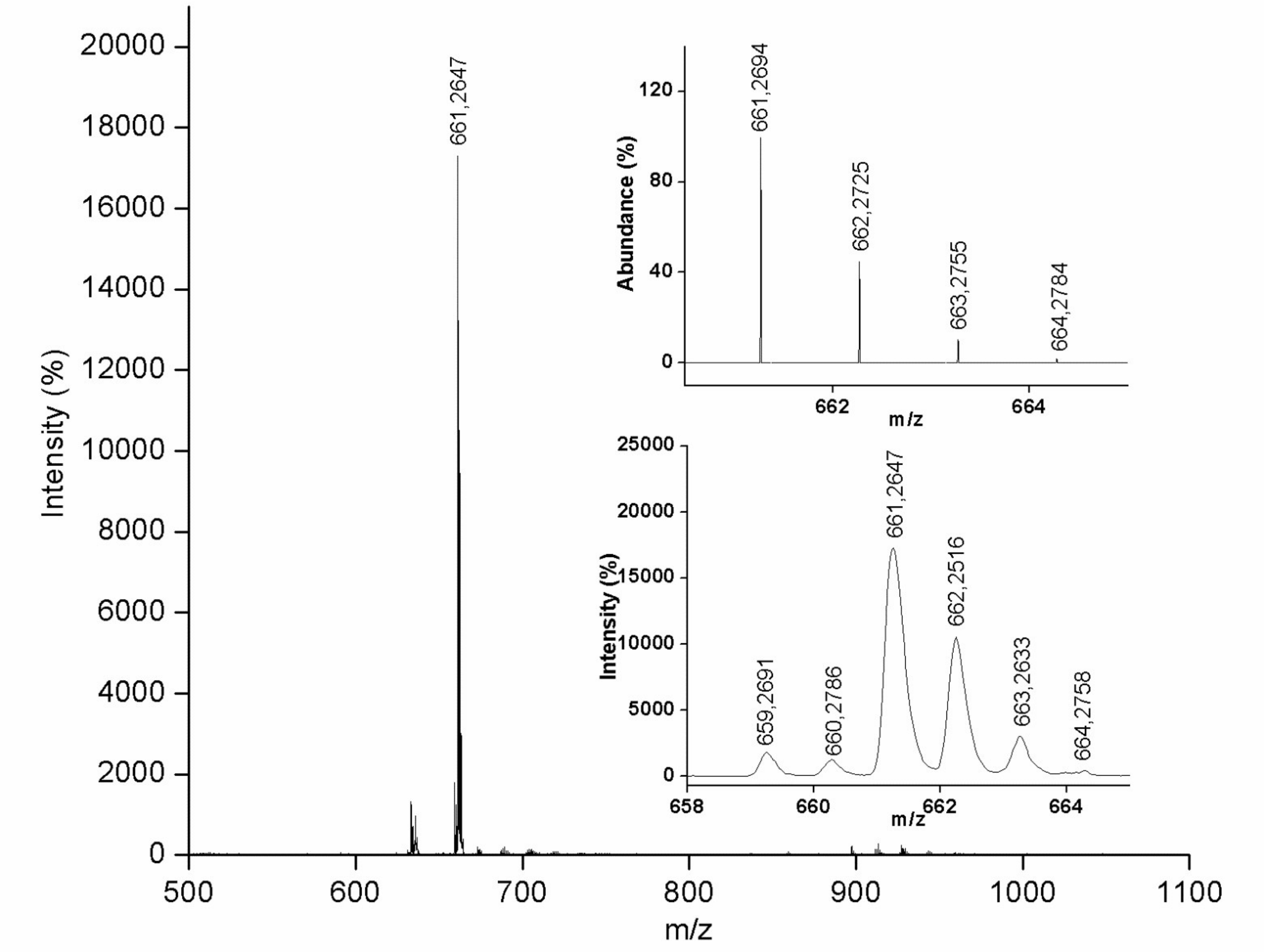

Figure S18: HR-MALDI-TOF spectrum of **5**

References

H. M. Osorio, S. Catarelli, P. Cea, J. B. Gluyas, F. Hartl, S. J. Higgins, E. Leary, P. J. Low, S. Martin, R. J. Nichols, J. Tory, J. Ulstrup, A. Vezzoli, D. C. Milan and Q. Zeng, *J. Am. Chem. Soc.,* **2015**, *137*, 14319.

2 a) L. T. Qu, Y. Liu, J. B. Baek and L. M. Dai, *ACS Nano,* **2010**, 4, 1321; b) A. L. M. Reddy, A. Srivastava, S. R. Gowda, H. Gullapalli, M. Dubey and P. M. Ajayan, *ACS Nano,* **2010**, *4*, 6337.

3. A. ONeil, U. Khan, P. N. Nirmalraj, J. Boland and J. N. Coleman, *J. Phys. Chem. C* , **2011**, *115*, 5422.
